# The consequences of a high-calorie diet background before calorie restriction on skeletal muscles in a mouse model

**DOI:** 10.18632/aging.203237

**Published:** 2021-06-24

**Authors:** Martin Maldonado, Jianying Chen, Yang Lujun, Huiqin Duan, Mazhar Ali Raja, Ting Qu, Tianhua Huang, Jiang Gu, Ying Zhong

**Affiliations:** 1 Chengdu Jinxin Institute of Reproductive Medicine and Genetics, Chengdu Jinjiang Hospital for Maternal and Child Health Care, Chengdu 610066, China; 2 Translational Medical Center, Second Affiliated Hospital of Shantou University Medical College, Shantou 515041, Guangdong, P.R. China

**Keywords:** calorie-intervention, skeletal muscle, sirtuins, mitochondria, adiponectin

## Abstract

The beneficial effects of calorie restriction (CR) are numerous. However, there is no scientific evidence about how a high-calorie diet (HCD) background influences the mechanisms underlying CR on skeletal muscles in an experimental mouse model.

Herein we present empirical evidence showing significant interactions between HCD (4 months) and CR (3 months).

Pectoralis major and quadriceps femoris *vastus medialis*, in the experimental and control groups, displayed metabolic and physiologic heterogeneity and remarkable plasticity, according to the dietary interventions.

HCD-CR not only altered genetic activation patterns of satellite SC markers but also boosted the expression of myogenic regulatory factors and key activators of mitochondrial biogenesis, which in turn were also associated with metabolic fiber transition.

Our data prompt us to theorize that the effects of CR may vary according to the physiologic, metabolic, and genetic peculiarities of the skeletal muscle described here and that INTM/IM lipid infiltration and tissue-specific fuel-energy status (demand/supply) both hold dependent-interacting roles with other key anti-aging mechanisms triggered by CR.

Systematic integration of an HCD with CR appears to bring potential benefits for skeletal muscle function and energy metabolism. However, at this stage of our research, an optimal balance between the two dietary conditions, where anti-aging effects can be accomplished, is under intensive investigation in combination with other tissues and organs at different levels of organization within the organ system.

## INTRODUCTION

The idea that reductions in calorie intake without malnutrition could slow aging, decrease age-related chronic diseases, and lengthen lifespan was postulated nearly a century ago by McCay and colleagues when they observed that rodents subjected to calorie restriction (CR) lived far longer than their *ad libitum*-fed counterparts [1].

CR rapidly alters the physiology and function of muscle stem cells (SC) in such a way that even short-term CR in rats that begins late in life (~18 months of age) has significant positive impacts in strengthening myogenic activity [2].

Muscle satellite cells in the adult skeletal muscles are characterized by the expression of the paired-domain transcription factors Pax7 [3] and Pax3 [4], the myogenic regulatory factors Myf5 and MyoD [5, 6], and the cluster of differentiation protein CD34 [7], among others. Of these, PAX7 is the canonical biomarker for satellite cells because it is particularly expressed in all quiescent and proliferating satellite cells across multiple species, including primates (humans and monkeys) and mice [3].

One of the beneficial effects of CR on satellite cell frequency in murine models is associated with metabolic rearranging that facilitates oxidative over glycolytic metabolism and is linked to pivotal modulators of mitochondrial mass and function, such as the NAD-dependent protein deacetylase SIRT1 [2].

SIRT1, the mammalian ortholog of Sir2 in yeast, is a nutrient-sensing and longevity factor that mediates the effects of dietary restriction in diverse species. In rodents, the expression of SIRT1 increases in response to prolonged CR in different tissues such as adipose, liver, kidney, and brain tissue [8, 9]. SIRT1 cross-talk and integrate signals with the energy-nutrient sensors AMPK, PGC-1α, and the mammalian target of Rapamycin (mTOR) [10–12] ensuring management and regulation of many cellular and metabolic processes, autophagy, and cell homeostasis.

CR has been extensively linked with mitochondria proliferation, biogenesis, dynamics, morphology, and reductions in oxidative stress [8, 13–15]. Oxidative stress induces telomere dysfunction, damage [16], and shortening of telomere to a critical length that can trigger aging and reduce life spans in mice and humans by mechanisms that involve the induction of persistent DNA damage response at chromosome ends [17]. However, CR has been suggested to synergize with telomerase expression, resulting in significant lifespan extension [18].

Moreover, adiponectin, a protein hormone encoded by the gene ADIPOQ in humans and mice and produced in adipose tissue, has been linked with CR. Indeed, CR appears not only to upregulate plasma adiponectin levels [19] but also appears to be associated with distinct metabolic states, suggesting that adipose tissue signaling is a suitable target for interventions to delay aging [20].

Herein, we attempt to correlate the above effects of CR, in two anatomically distinct skeletal muscles, with a high-calorie diet (HCD) background. We present evidence for the first time of interactions between CR and HCD and discuss different genetic mechanisms and pathways that may interact to modulate physiologic and metabolic changes elicited by the dietary interventions.

## RESULTS

### Development and characterization of the experimental animal model

**Figure 1:**
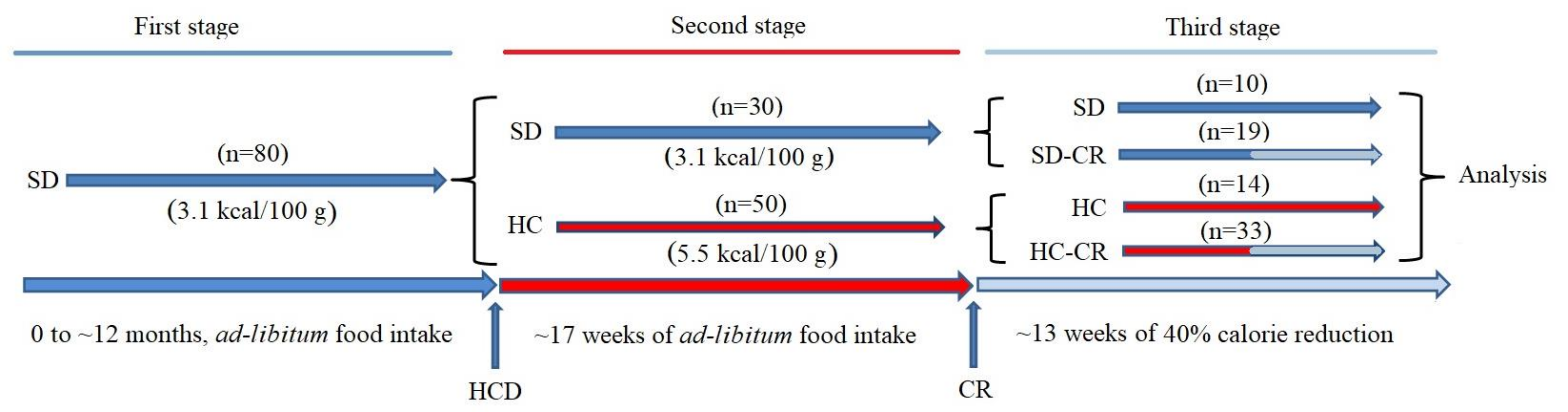
**Generation of the experimental animals.** CD-1 mice were fed *ad libitum* standard food (First stage) until they reached 12 months of age. Animals were separated into a group fed *ad libitum* standard food (SD) and *ad libitum* HCD (HC), for 17 weeks (Second stage). SD and HC animals were subdivided into control and experimental groups (Third stage). The experimental groups (SD-CR and HC-CR) were subjected to calorie restriction (CR) for 13 weeks before sacrifice.

**Figure 2:**
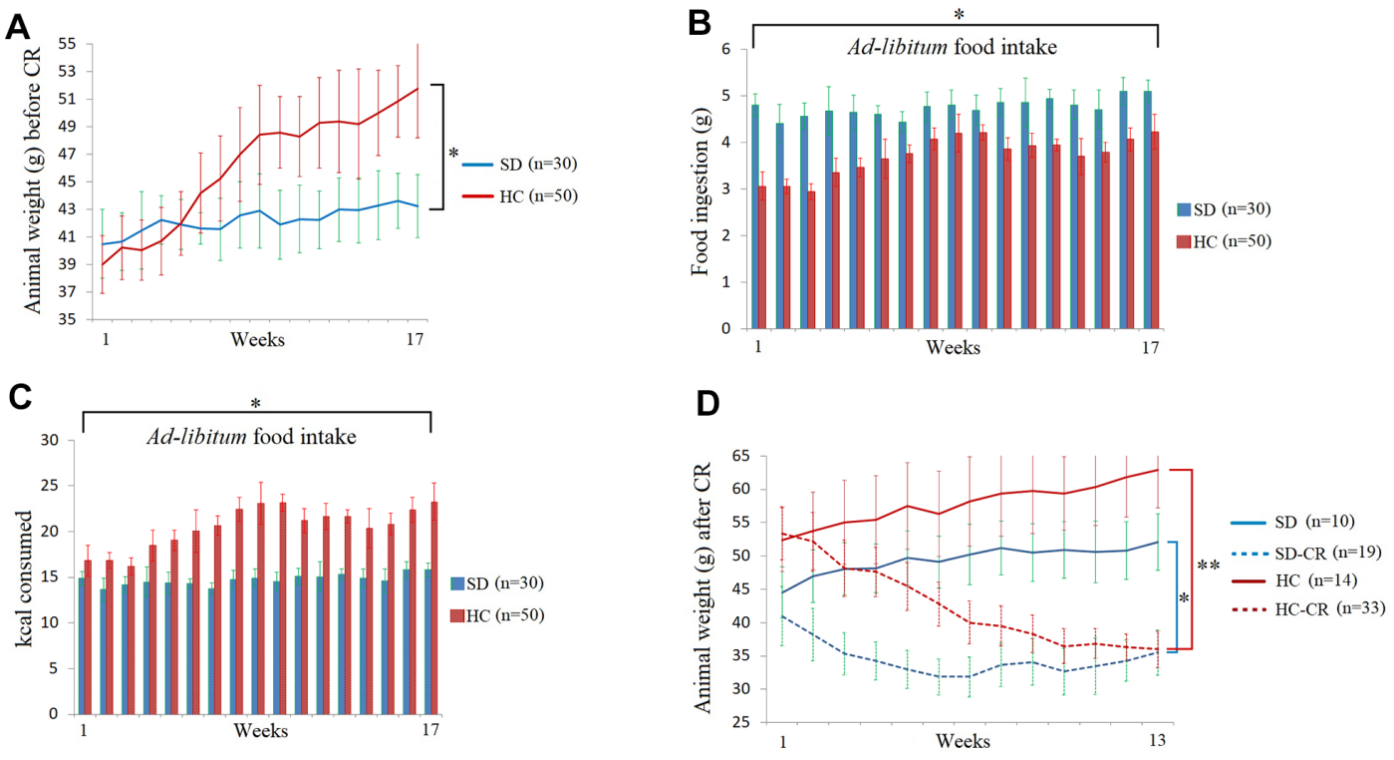
**Food and calorie consumption during the development of the experimental animal model.** The body weights of the average of the animal groups before calorie restriction (CR) (**A**). Food ingestion of the average of the animal groups expressed in grams before CR (**B**). Kcal consumption of the average of the animal groups before CR (**C**). The animal weights expressed in grams during the CR period (**D**). The data are the mean ± s.d. *P < 0.05; **P < 0.01 vs SD, unless otherwise specified.

The steps for the generation of animal models can be seen in Figure 1 and Materials and Methods. Mice fed *ad libitum* HCD showed significant increases in body weight (Figure 2A). Interestingly, the animals subjected to HCD (HC) presented reduced daily volumes of ingested food (Figure 2B) compared to those fed the standard 3.1 kcal/g diet *ad libitum* (SD). Nevertheless, due to food composition, the calorie intake of the HC was significantly higher than that of its control counterpart (Figure 2C).

At the end of the CR period, the SD-CR and HC-CR showed no bodyweight differences between them (Figure 2D).

**Figure 3:**
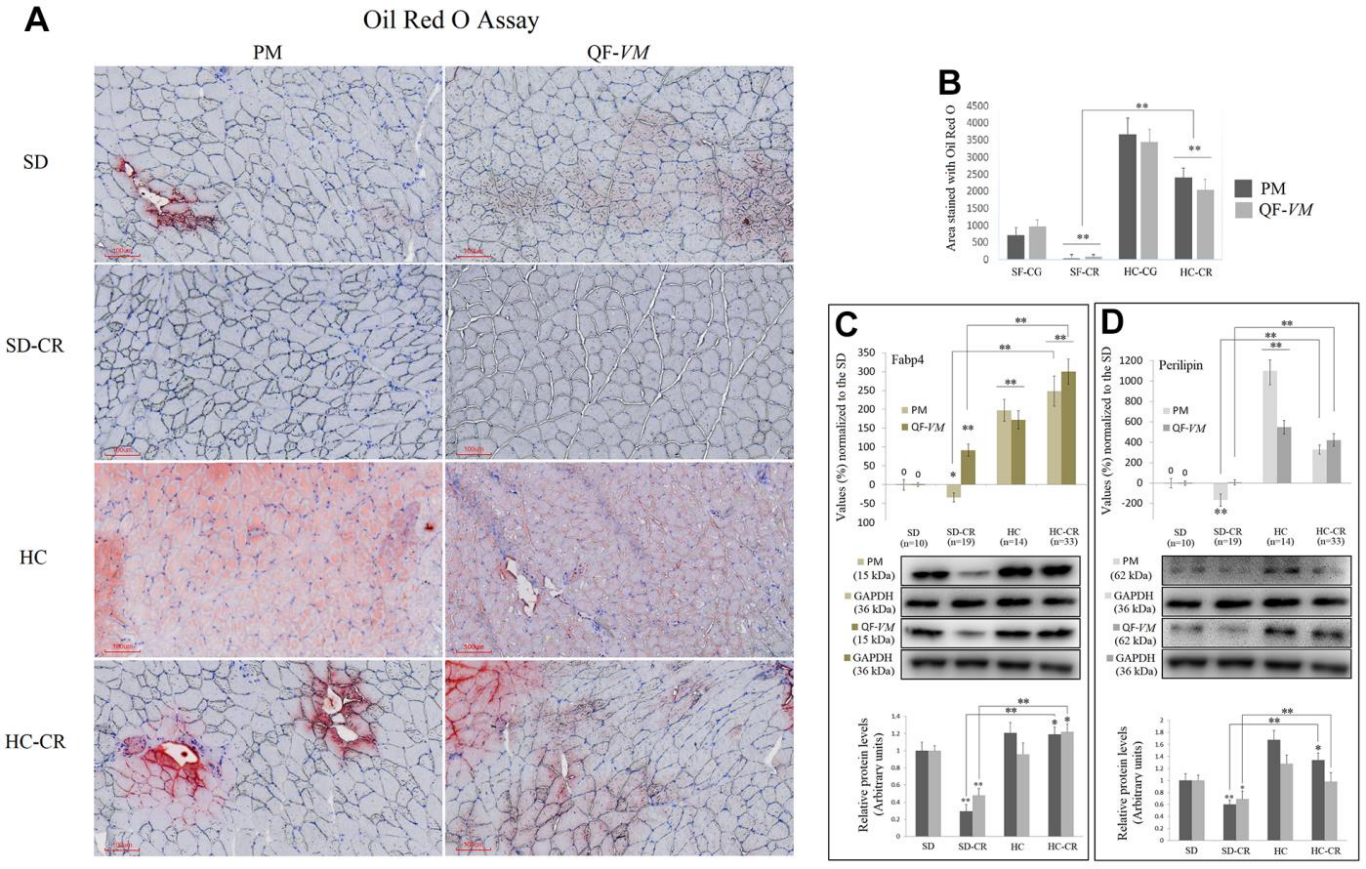
**Lipid content determination in PM and QF-*VM* of the experimental animal model.** Representative images of lipid content stained with the oil red O technique (**A**). Scale bar: 100 μm. SD (n=6), SD-CR (n=8), HCD (n=9), and HCD-CR (n=12). The total area stained with Oil red O (**B**). Fabp4 (**C**) and Perilipin (**D**) were used to predict inter- (INTM) and intra-myocellular (IM) fat infiltration. Fabp4 and Perilipin were analyzed by qPCR (mRNA) and expressed as %, the SD value was set to 0, and the compared samples were normalized to this level. Positive % values represent upregulation. Negative values represent downregulation. Protein expression of Fabp4 and Perilipin was obtained by Western Blot analysis and quantified with Image Lab 6.1 software. SD expression was set to 1 and the relative protein levels were normalized as a ratio of GAPDH expression. The data are the mean ± s.d. *P < 0.05; **P < 0.01 vs SD, unless otherwise specified.

To determine total lipid content in PM and QF-*VM*, we used the Oil Red O technique (Figure 3A, 3B). Moreover, we analyzed the gene and protein expression of the adipose-type cytoplasmic fatty acid-binding protein (Fabp4, Figure 3C), used to predict inter- (INTM) and intra-myocellular (IM) fat infiltration [21], and Perilipin (Figure 3D), the most abundant phosphoprotein on adipocyte lipid droplets [22].

The combined data showed that the HC-CR contains higher amounts of lipid content compared to that in the SD-CR, even though, as mentioned before, the average body weight of the two experimental groups was almost identical at the end of the CR period.

Due to the small size of the animals, we did not distinguish INTM and IM adiposity; a limitation that has been already observed in other available studies of IM adipogenesis in mice [23].

**Figure 4:**
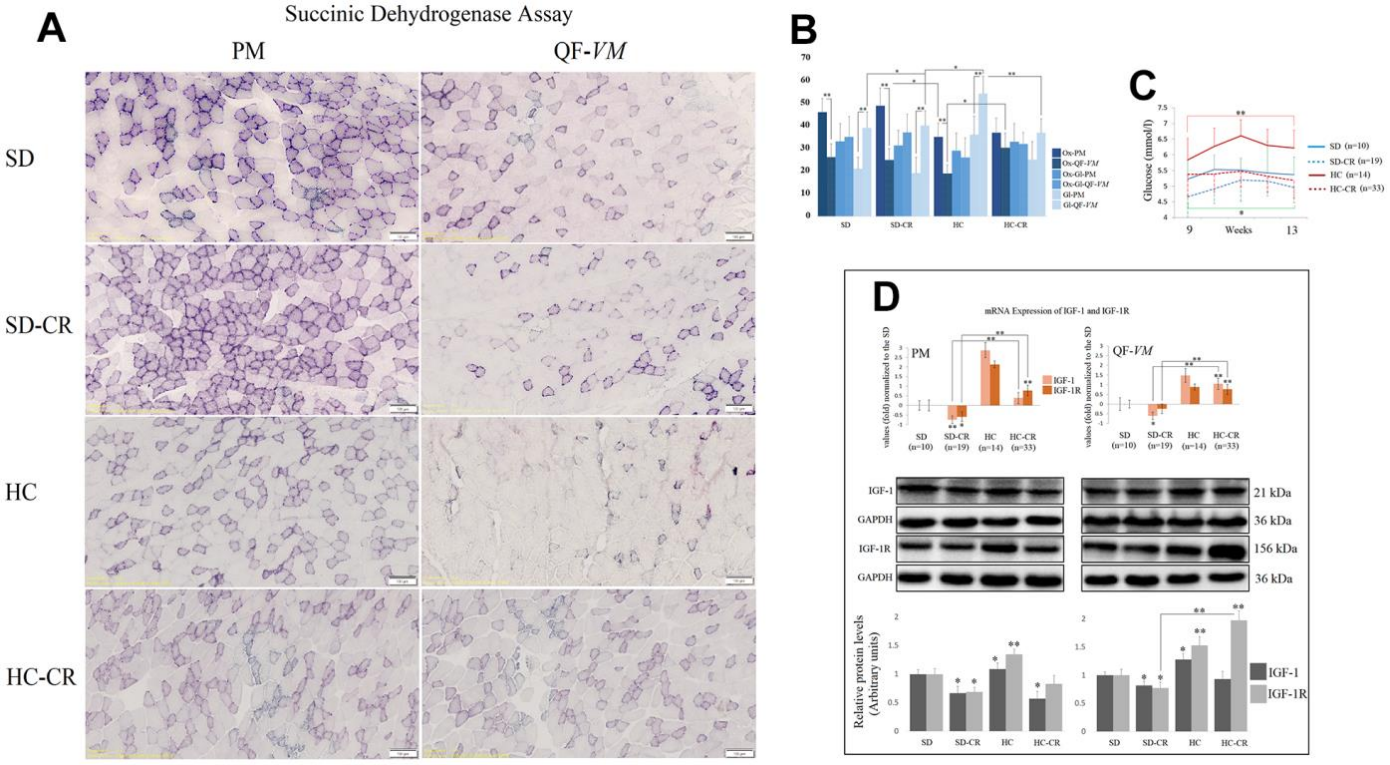
**Metabolic characterization of PM and QF-*VM* in the experimental animal model.** Representative images of SDH staining in muscle fibers of PM and QF-VM in the different groups (**A**). Scale bar: 100 μm. SD (n=6), SD-CR (n=8), HCD (n=9), and HCD-CR (n=12). The total area stained with Oil red O (**B**). Dark stained fibers were classified as oxidative (Ox); intermediate stained fibers were classified as oxidative-glycolytic (Ox-Gl); and very lightly or non-stained fibers were classified as glycolytic (Gl). Comparison of the blood glucose levels of mice during week 9 to 13 of the dietary restriction (**C**). IGF-1 and IGF-1R were analyzed by qPCR and Western Blot analysis (**D**). mRNA was expressed as fold, the SD value was set to 0, and the compared samples were normalized to this level. Positive values represent upregulation. Negative values represent downregulation. Protein expression of IGF-1 and IGF-1R was obtained by Western Blot analysis and quantified with Image Lab 6.1 software. SD expression was set to 1 and the relative protein levels were normalized as a ratio of GAPDH expression. The data are the mean ± s.d. *P < 0.05; **P < 0.01 vs SD, unless otherwise specified.

To characterize the metabolic status of the experimental and control groups, we determined the oxidative and glycolytic muscle fiber properties of PM and QF-*VM* with the SDH technique (Figure 4A, 4B).

Overall, the SD and SD-CR in PM displayed extensive areas of oxidative fibers, while the animals fed HCD (HC) showed fewer clusters of the oxidative type (vs SD-CR) and denser areas of intermediate and glycolytic fibers. Although not statistically significant (when the metabolic fiber types were analyzed individually), the HC-CR-PM presented a mild transition from glycolytic to oxidative activity seen by the increase in oxidative and intermediate fibers and a decrease in the glycolytic type.

In addition, QF-*VM* in the different groups appeared to have denser areas of glycolytic fibers than those seen in PM, and the HC presented significantly higher amounts of glycolytic fibers than the rest of the groups analyzed. However, when these animals with an HCD background (HC) were subjected to CR, we observed a clear transition from glycolytic to the oxidative phenotype.

Serum glucose levels were measured after 8 h of fasting conditions (Figure 4C). The data showed that mice subjected to CR (SD-CR and HC-CR) presented improved blood glucose levels (compared to *ad libitum*-fed mice), eight weeks after the beginning of the dietary restriction.

We also analyzed the insulin-like growth factor 1(IGF-1) and its receptor IGF-1R (Figure 4D) because of its wide range of physiological and cellular functions, one of which is the regulation of glucose metabolism. We chose tissue-specific expression rather than circulating levels mainly because the technique allows the comparison of the two skeletal muscles and because intrinsic secretion of muscle IGF-1, rather than circulating plasma IGF-1, is a key element for switching on anabolic pathways [24].

Our results showed that CR decreased mRNA and protein levels of IGF-1 and IGF-1R (IGF-1R-SD-CR-QF-*VM*, not statistically significant) in the animals previously fed standard food (SD-CR), in both PM and QF-*VM*. Although CR decreased IGF-1 protein levels in HC-CR-PM, we did not observe significant changes in IGF-1 translational expression in the HC-CR-QF-*VM*. This was also accompanied by a marked increase in IGF-1/IGF-1R mRNA and IGF-1R protein expression. Furthermore, the HC animals presented IGF-1 and IGF-1R mRNA and protein upregulations in both skeletal muscles.

### Expression of satellite SC markers in mice subjected to CR

**Figure 5:**
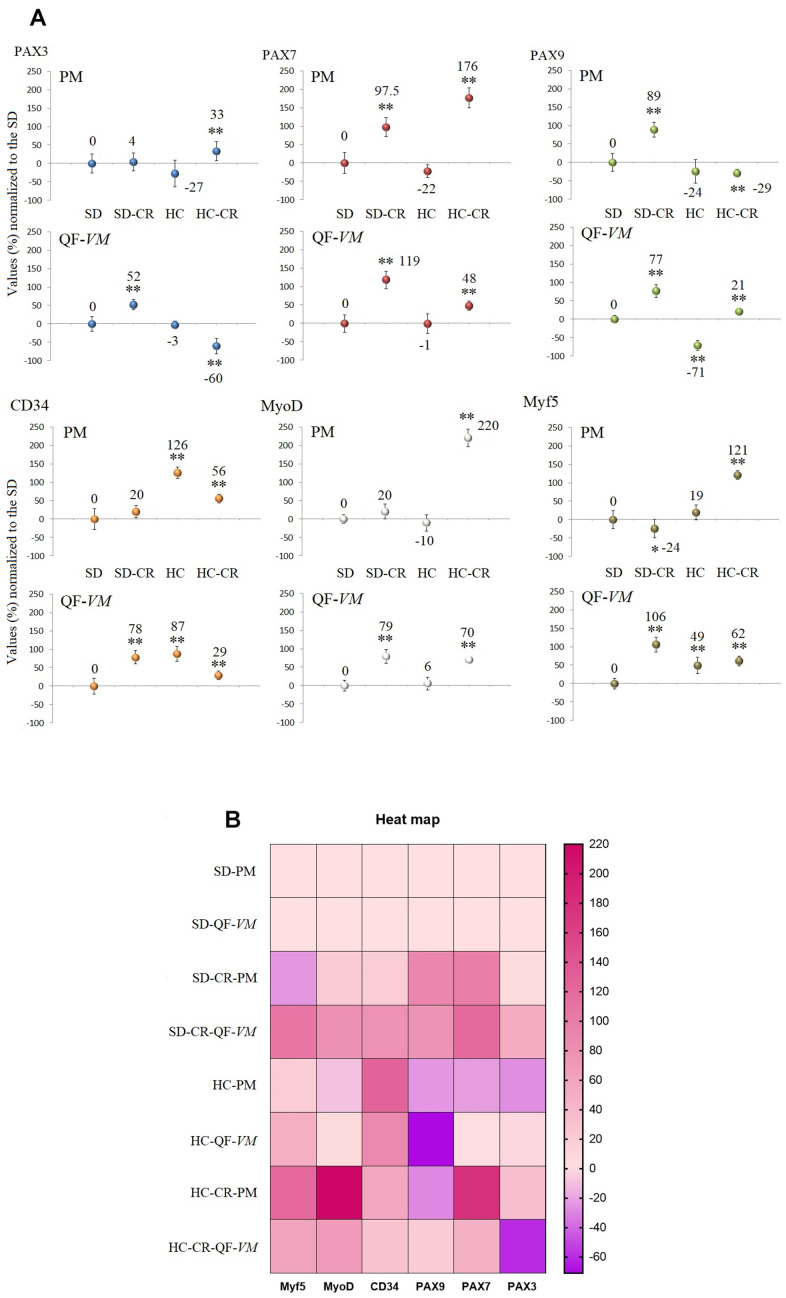
**Gene expression of satellite markers.** cDNA from PM and QF-*VM* was obtained with a Takara RR047Q kit. qPCR was performed with 100 ng of target DNA to measure PAX3, PAX7, PAX9, CD34, MyoD, Myf5, and 18s rRNA (reference gene) expression (**A**). For each gene expressed as percentage (%), the SD value was set to 0, and the compared samples were normalized to this level. Positive values represent upregulation. Negative values represent downregulation. Each marker was analyzed with SYBR Green fluorescence detection, and the transcript levels were normalized to those of the endogenous control 18s rRNA. The data are the mean ± s.d. *P < 0.05; **P < 0.01 vs SD. A hierarchical clustering illustration for the up/down-regulation of genes analyzed from RT-PCR array data. The right bar depicts the colors for upregulation and downregulation expressed as a % (**B**).

We analyzed the satellite cell biomarkers PAX3, PAX7, PAX9, CD34, MyoD, and MyF5 from the QF-*VM* and the PM tissues in the different groups by qPCR (Figure 5A, 5B). After 13 weeks of CR, we observed 2 different and independent phenomena: First, the gene expression patterns differed between the muscles analyzed: Although the SD-CR presented an overall tendency toward overexpression, only PAX7 (97%, + 26) and PAX9 (89%, + 20) were significantly upregulated in PM (compared to those in the SD), whereas in QF-*VM* all the satellite transcripts were significantly increased. Furthermore, the animals in the HC-CR-PM presented higher expression levels in PAX3 (33%, + 7), PAX7 (176%, + 27), CD34 (56%, + 12), MyoD (220%, + 24), and Myf5 (121%, + 12), whereas in the HC-CR-QF-*VM* only PAX9 (21%, + 4), MyoD (70%, + 8) and Myf5 (62%, + 13) were upregulated.

Second, the gene expression patterns differed between the animals exposed to CR with different alimentary backgrounds (SD-CR and HC-CR), as PAX9-PM was strongly upregulated (89%, + 20) in the SD-CR (compared to those in the SD) while significantly downregulated to -29%, + 10 in the HC-CR (compared to those in the SD). Other examples are PAX3-QF-*VM* (SD-CR upregulation to 52%, + 14; HC-CR downregulation to -60%, + 21) and Myf5-PM (SD-CR downregulation to -24%, + 6; HC-CR upregulation to 121%, + 12).

The gene expression of satellite and other markers, expressed as a percentage (%) or fold change values can be found in the Supplementary Table 4.

### Mitochondrial DNA copy numbers and mitochondrial biogenesis-related activators in mice subjected to CR

**Figure 6:**
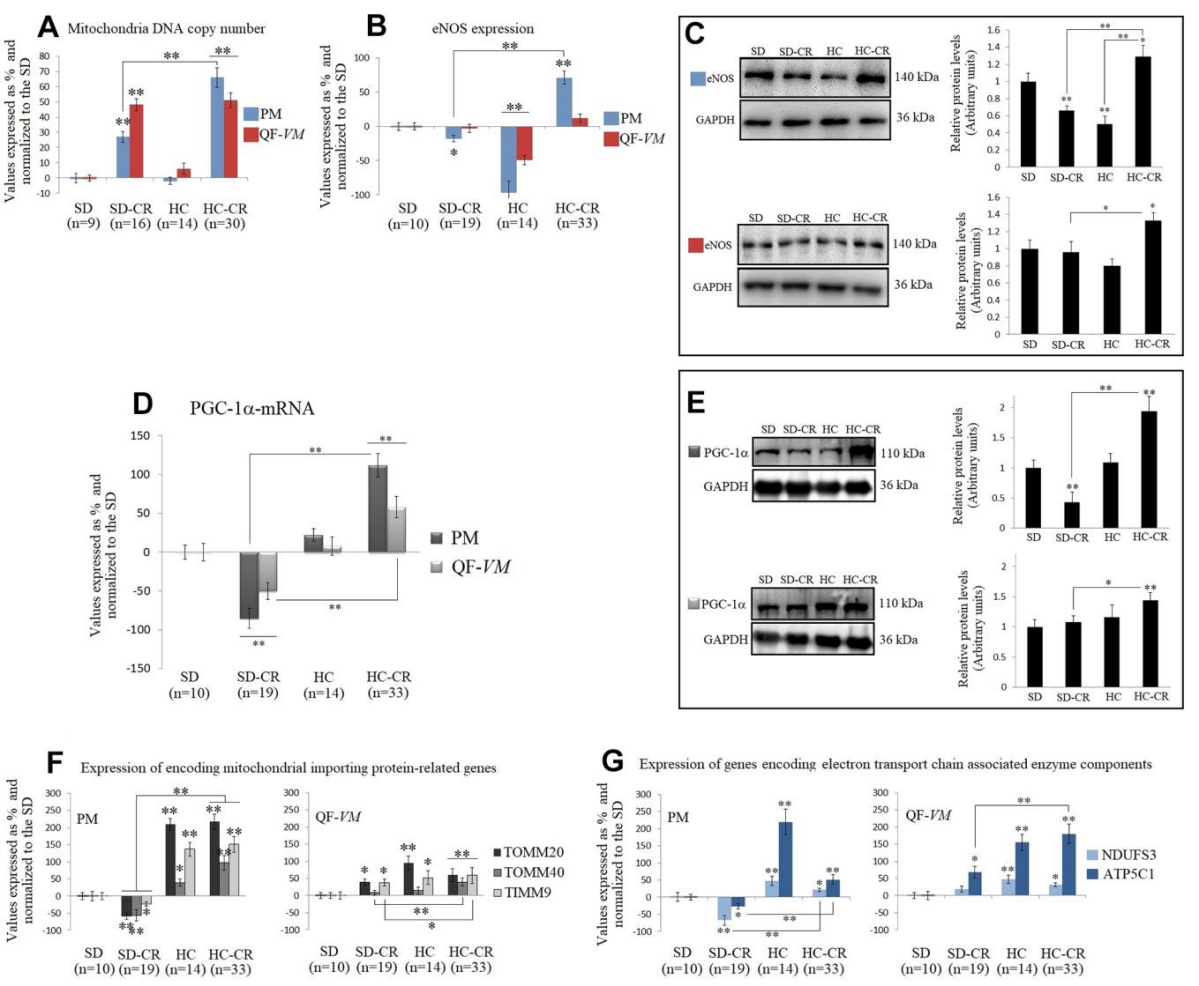
**Mitochondrial DNA copy numbers and the expression of mitochondrial biogenesis-related activators in mice subjected to CR.** Mitochondrial DNA copy number (**A**) was analyzed with the Detroit-Mouse-mt-DNA analysis kit (Supplementary Material, 1). For each group, the value was expressed as a percentage (%); the SD value was set to 0, and the rest of the groups were normalized to this level for comparison. eNOS (**B**, **C**) and PGC-1α (**D**, **E**) were analyzed at the transcriptional and translational levels. TOMM20, TOMM40, TIMM9 (**F**), and NDUFS3 and ATP5C1 (**G**), were analyzed at the transcriptional level. For the qPCR assay, each primer was analyzed with SYBR Green fluorescence detection and the transcript levels, expressed as a %, were normalized to those of the endogenous control 18s rRNA. Protein expressions were obtained by Western Blot analysis and quantified with Image Lab 6.1 software. SD expression was set as 1 and the relative protein levels were normalized as a ratio of GAPDH expression. The data are the mean ± s.d. *P < 0.05; **P < 0.01 vs SD, unless otherwise specified.

The mitochondrial DNA copy numbers in the groups subjected to CR (the SD-CR and HC-CR) were increased in PM tissue by 28%, + 3.5, and 66% + 6.4 respectively (vs SD-PM), and in QF-*VM* tissue by 48.1 + 4 and 51.2% + 4.9 respectively (vs SD-QF-*VM*; Figure 6A). Our results appear to agree with scientific data supporting the idea that CR induces mitochondrial proliferation in rodents [16, 8].

Then, we assayed the mRNA and protein expression of eNOS (Figure 6B, 6C), which has important roles in the regulation of mitochondrial function and induction of mitochondrial biogenesis [25], and PGC-1α (Figure 6D, 6E), a transcriptional co-activator, and also a master regulator of mitochondrial biogenesis [26, 27].

Interestingly, eNOS and PGC-1α in the HC-CR showed similar increased expression patterns (except for eNOS-QF-*VM* mRNA) compared to those found in the SD-CR, while the SD-CR-PM presented significant downregulations (vs SD).

We also measured the transcript levels of TOMM20, TOMM40, and TIMM9 (Figure 6F), which are responsible for encoding mitochondrial importing proteins, and NDUFS3 and ATP5C1 (Figure 6G), which encode electron transport chain associated enzyme components, because they reflect function and biogenesis rather than abundance [28].

Similar to the previous patterns found in eNOS and PGC-1α, TOMM20, TOMM40, TIMM9, NDUFS3, and ATP5C1 were significantly upregulated in the HC-CR (except for TOMM20-QF-*VM*, and NDUFS3- QF-*VM*; vs SD-CR).

The SD-CR-QF-*VM*, unlike SD-CR-PM, presented overexpression of TOMM20, TIMM9, and ATP5C1 in (vs SD), while eNOS and PGC-1α remained unchanged (vs SD).

These findings suggest that although elevated amounts of mitochondria DNA content were found in both CR-treated groups, only the HC-CR and may present improved mitochondrial biogenesis and function.

### Telomerase activity was increased in animals subjected to CR

**Figure 7:**
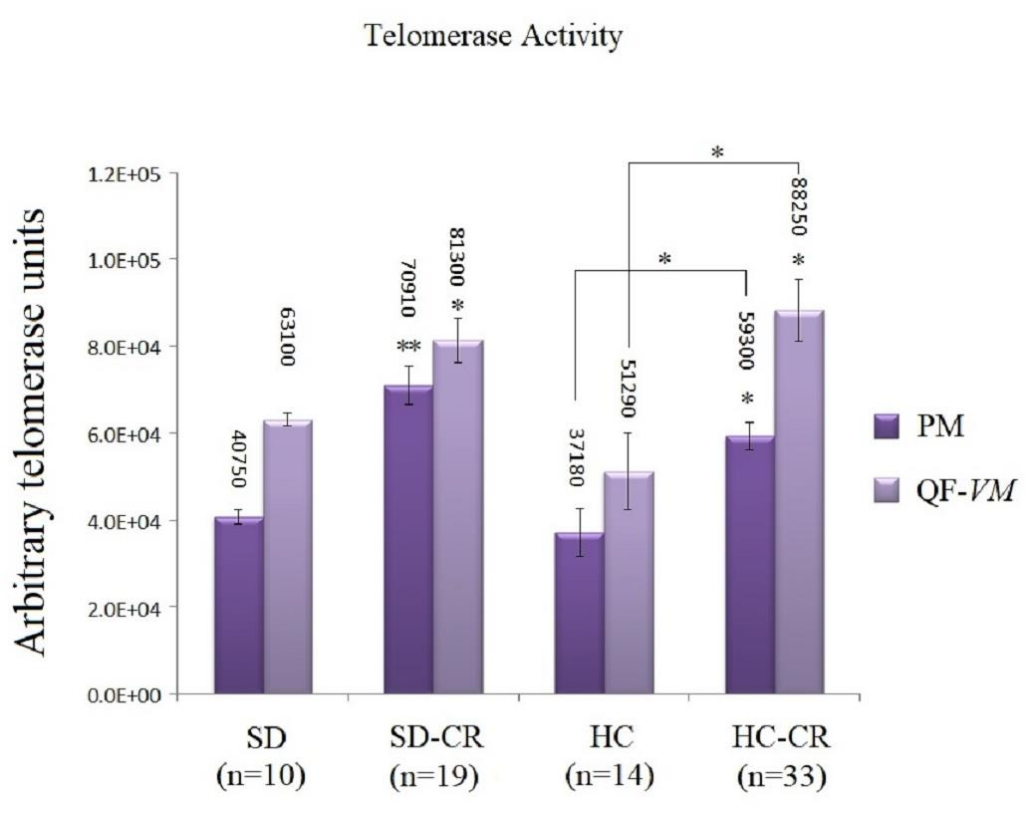
**Telomerase activity.** Telomerase activity was assayed with a TRAPeze RT Telomerase Detection Kit (Millipore) for fluorometric detection and real-time quantification in pectoralis major (PM) and quadriceps femoris v*astus medialis* (QF-*VM*). The telomerase values are arbitrary units relative to the TSR8 amplification, according to the assay kit protocol (Supplementary Material 2). The data are the mean ± s.d. *P < 0.05; **P < 0.01 vs SD, unless otherwise specified.

The groups subjected to CR (SD-CR and HC-CR) expressed significantly higher telomerase activity in both PM and QF-*VM* tissue compared to the CGs (Figure 7), evidence that suggests a possible interaction between these two major anti-aging mechanisms. Moreover, the PM tissue appeared to have lower telomerase activity than that found in QF-*VM*.

### CR and the AMPK-SIRT1-mTOR network

AMPKs and SIRT1, as well as mTOR (a central controller of cell growth and proliferation), are involved in the regulation of many cellular and metabolic processes. The cross-talk among these three enzymes occurs at several levels and the action of one may be modulated by the actions of the other(s) in some cases [13].

**Figure 8:**
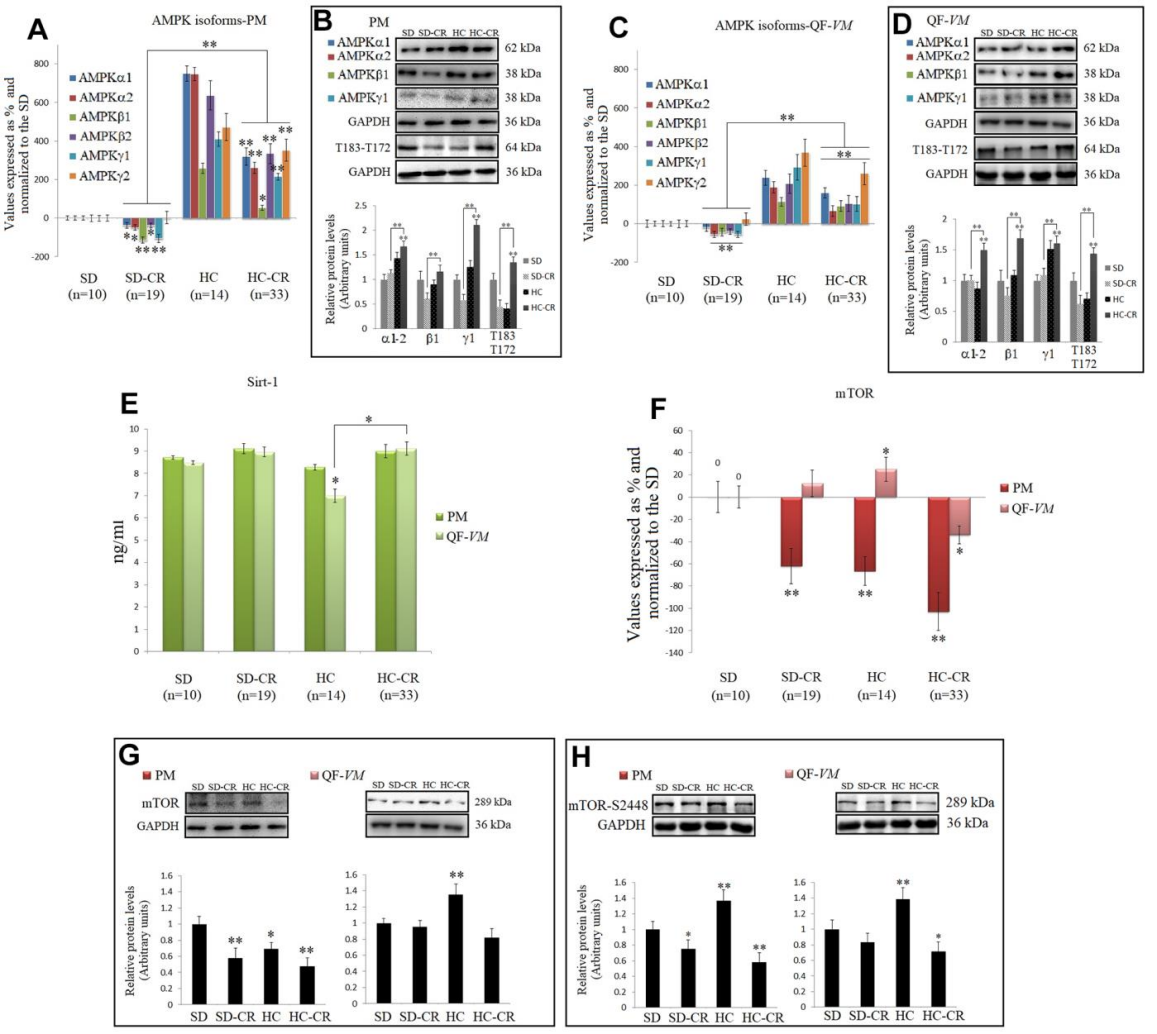
**CR and the AMPK-SIRT1-mTOR network.** AMPKs were analyzed by qPCR (mRNA levels) and Western Blot (protein levels) in pectoralis major (PM) (**A**, **B**) and quadriceps femoris *vastus medialis* (*QF*-*VM*) (**C**, **D**). SIRT1 was analyzed in PM and QF-*VM* (**E**) with the Mouse NAD-Dependent Deacetylase Sirtuin-1 (SIRT1) ELISA Kit (CUSABIO) for quantitative determination, according to the assay kit protocol (Supplementary Material 3). Values are expressed in ng/ml. mTOR mRNA in PM and QF-*VM* (**F**). Immunoblot results and protein expression of mTOR (**G**) and mTOR-S2448 (**H**) in PM and QF-*VM*. For the qPCR assay, each primer was analyzed with SYBR Green fluorescence detection and the transcript levels, expressed as a %, were normalized to those of the endogenous control 18s rRNA. Protein expressions were obtained by Western Blot analysis and quantified with Image Lab 6.1 software. SD expression was set as 1 and the relative protein levels were normalized as a ratio of GAPDH expression. The data are the mean ± s.d. *P < 0.05; **P < 0.01 vs SD, unless otherwise specified.

CR is well known to increase AMPKs and SIRT1 expression in different tissues and species [29, 9], so we analyzed the mRNA and protein levels of AMPK, T183-T172, and SIRT1 endogenous levels (Figure 8A–8E).

Although we presented mRNA and protein data of the different isoforms of AMPK, we focused on T183-T172 as AMPK becomes activated when phosphorylation takes place at T183 (AMPK α1, phosphorylated at threonine 183) and T172 (AMPK α2, phosphorylated threonine 172).

Our data showed higher AMPK expressions and AMPK activity (T183-T172) in the HC-CR than those found in the SD-CR, in PM (Figure 8A, 8B), and QF-*VM* (Figure 8C, 8D). Furthermore, SIRT1, which followed similar expression patterns in the two muscles analyzed, was slightly increased in the animals subjected to CR, but the values (ng/ml) were not statistically significant (Figure 8E).

AMPK and SIRT1 are known to negatively regulate mTOR [30, 31], thus we checked mTOR (transcript, Figure 8F; protein, Figure 8G) expression, and the protein expression of mTOR phosphorylated at serine 2448 (mTOR-s2448; Figure 8H), as mTOR complex 1 (mTORC1–regulatory associated protein of mTOR (Raptor) positive, primarily phosphorylated at s2448 is a key regulator of skeletal muscle growth and cell proliferation through activation of mRNA translation/protein synthesis, and regulation of autophagy [32].

mTOR mRNA levels were significantly downregulated in PM (SD-CR -62% + 16; HC -66.5 + 13; HC-CR -103% + 22) and in the QF-*VM*, only the HC-CR was downregulated (-34% + 8). At the protein level, mTOR-HC-CR was significantly lowered compared to that in the SD-CR in PM (* < 0.05) and SD (PM, **<0.01; QF-*VM*, *<0.05).

The analysis of the mTORC1 complex showed activation in HC-PM and QF-VM (PM, **<0.01; QF-*VM*, **<0.01). On the other hand, the groups exposed to CR presented lower mTOR activity (PM-SD-CR, *<0.05; PM-HC-CR, **<0.01; QF-*VM*-HC-CR, *<0.05; vs SD). However, QF-*VM*-SD-CR displayed no statistical differences, vs SD.

### Adiponectin and adiponectin-receptors in mice subjected to CR

**Figure 9:**
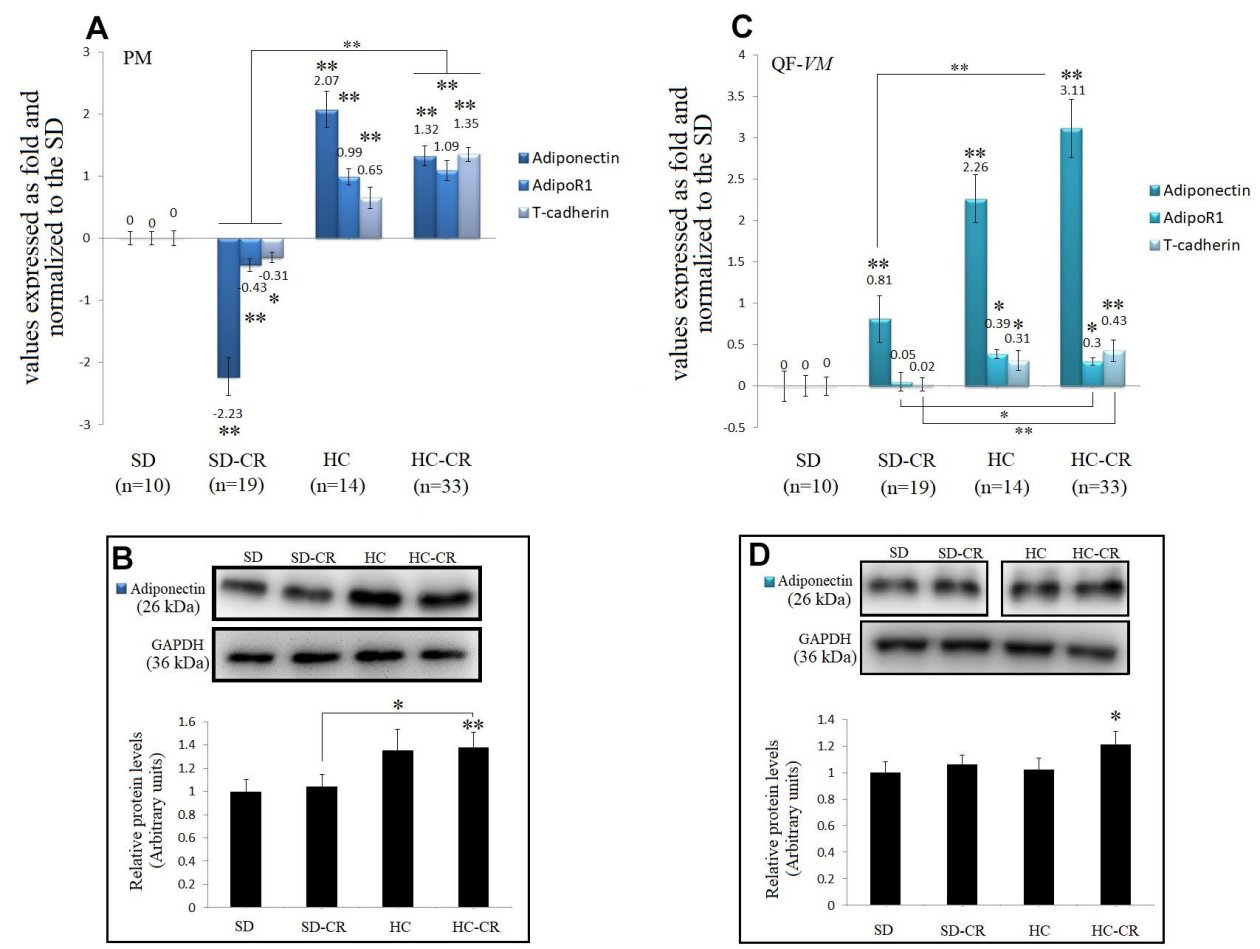
**Expression of Adiponectin and Adiponectin-receptors.** The mRNA levels of adiponectin, AdipoR1, and T-cadherin in pectoralis major (PM) (**A**). Immunoblot results and protein expression of adiponectin in PM (**B**). mRNA levels of adiponectin, AdipoR1, and T-cadherin in quadriceps femoris *vastus medialis* (*QF*-*VM*) (**C**). Immunoblot results and protein expression of adiponectin in QF-*VM* (**D**). Adiponectin, AdipoR1, T-cadherin, and 18s rRNA (reference gene) transcripts were analyzed by qPCR. Significant differences between groups are expressed as fold change values. For each gene expressed as a fold change, the SD value was set to 0, and the compared samples were normalized to this level. Positive values represent upregulation. Negative values represent downregulation. The fold changes are defined directly in terms of ratios. Each marker was analyzed with SYBR Green fluorescence detection, and the transcript levels were normalized to those of the endogenous control 18s rRNA. Protein expression of adiponectin in PM and QF-*VM* was obtained by Western Blot analysis and quantified with Image Lab 6.1 software. SD expression was set to 1 and the relative protein levels were normalized as a ratio of GAPDH expression. The data are the mean ± s.d. *P < 0.05; **P < 0.01 vs SD, unless otherwise specified.

The adipocyte-specific hormone and its receptors AdipoR1and T-cadherin were examined in PM (Figure 9A, 9B) and QF-*VM* tissues (Figure 9C, 9D).

Adiponectin mRNA expression was drastically increased not only in the animals exposed to CR (except for the SD-CR-PM, -2.23- + 0.3-fold) but also in the HC, in both muscle tissues. At the translational level, adiponectin also presented higher expression in the HC-CR compared to that in the SD-CR (Figure 9B, 9D), however, the differences were not significant in QF-*VM*.

Moreover, gene profiling of adiponectin in the HC-CR revealed positive correlations with the overexpression of the adiponectin receptors AdipoR1 (PM: *r*=0.77; QF-VM: *r*=0.66,) and T-cadherin (PM: *r*=0.79; QF-VM: *r*=0.72) in both tissues. Furthermore, the under-expression of adiponectin found in the SD-CR-PM was also strongly correlated with AdipoR1 (*r*=0.76) and T-cadherin (*r*=0.73) (Figure 9A).

## DISCUSSION

In this study, we have examined the effects of an HCD background in two skeletal muscles from distant anatomical locations, in mice subjected to CR. In terms of the physiologic, metabolic, and phenotypic characteristics, the skeletal muscles in the experimental and control groups presented not only differences in the amounts of lipid infiltration but also exhibited discrepancies in terms of metabolic fiber properties, as well as metabolic fiber transition, according to the dietary interventions. PM enzymatic activity was predominantly oxidative in the SD whereas QF-V*M* presented more extensive areas of the glycolytic type. HCD induced fiber transition to the glycolytic phenotype in both muscles (HC groups), which was consistent with other reports showing inverse relationships between adiposity and the oxidative/type 1 muscle fiber [33–36]. Moreover, HC-CR increased the number of oxidative/ intermediate fibers in PM and QF-*VM*. These results were associated with the protein and mRNA-overexpression of the transcriptional co-activator PGC-1α (PM-HC-CR, **<0.01; QF-*VM*-HC-CR, **<0.01) that has been reported to increase the proportion of red/oxidative type I fibers [37].

Numerous reasons such as nutrition, hormone levels, exercise, and other external environmental insults have been reported to affect muscle fiber type transition [38, 39], and the CR**-**mimetic Resveratrol has also been reported to induce myofiber type transition from glycolytic to oxidative in skeletal muscles [40–42].

Furthermore (except for HC-CR-QF-*VM*), CR decreased IGF-1 and IGF-1R protein expressions, consistent with other previous reports in rodents [43–45]. Nevertheless, IGF-1 translational levels in the HC-CR-QF-*VM* showed no differences than those found in the SD and displayed significantly higher expressions of IGF-1R. These results could be explained at least in part, by the effect of IGF-1 on mitochondrial mass via increased transcriptional activities of key factors involved in mitochondrial biogenesis like PGC-1α [46].

Prior studies in the rodent skeletal muscles [47, 2] suggested that short-term and lifelong CR increase and maintain the satellite cell number and function which normally declines with age, but whether short-term CR in combination with HCD in otherwise healthy aged animals reshapes the expression of specific markers of satellite SCs have not yet been addressed.

Due to the experimental design, in this work satellite SCs were not isolated, and the mRNA data represent the expression of the whole muscle tissue. Nevertheless, the two observed independent events already stated in the results section (contrasting gene expression patterns differed between the muscles analyzed; different gene expression patterns between the animals exposed to CR with different alimentary backgrounds) may reflect the dynamics of the gene expression in the satellite SC pools. That being said, isolation and characterization of satellite SCs under the same experimental conditions would be necessary to confirm this hypothesis.

Concerning the analysis of mitochondrial function, biogenesis, and number, CR promoted mitochondrial abundance by increasing the amounts of mtDNA [48] in both CR-treated groups in PM and QF-*VM*, regardless of the metabolic fiber type composition/activity, as the slow-twitch oxidative fibers (predominantly found in PM) contain many more mitochondria than the glycolytic type (predominantly found in QF-*VM*). The aerobic metabolism, which consumes O_2_, occurs in the mitochondria, thus the levels of oxidative enzymes in the different muscle fibers reflect the relative abundance of mitochondria, as determined by electron microscopy [49].

Animals fed HCD previous to CR (HC-CR-PM and QF-*VM*) displayed increased expressions of the key regulators of mitochondrial biogenesis eNOS, PGC-1α, and AMPK (discussed in the *AMPK-SIRT1-mTOR network*).

On the other hand, mice fed standard food previous to CR (SD-CR) showed an overall reduction in the above-mentioned regulators of mitochondrial biogenesis (vs HC-CR), and part of these inconsistencies could be attributed to the processes involved in fiber transition to the oxidative phenotype, seen in the HC-CR-PM and -QF-*VM* (marked plasticity in the glycolytic/ intermediate QF-*VM*); phenotypic changes that we did not observe in the animals treated with CR alone (SD-CR).

Moreover, differences between muscles, in the SD-CR, were also evident. The SD-CR-PM (vs SD and HC-CR) displayed reduced expressions of eNOS (mRNA, Figure 6B; protein levels, Figure 6C) and PGC-1α (mRNA, Figure 6D; protein levels, Figure 6E), TOMM20, TOMM40, TIMM9, NDUFS3, and ATP5C1 mRNA (Figure 6F, 6G). These peculiarities might be attributed at last in part, to the contrasting metabolic fiber activity (PM vs QF-*VM*), determined by the SDH technique (PM: Oxidative/intermediate/glycolytic; QF-*VM*: Glycolytic/intermediate/oxidative).

The CR-treated groups also presented mRNA upregulations of the myogenic regulatory factors (MRFs) MyoD and Myf5, except for the SC-CR-PM. Although little is known about Myf5, MyoD directly binds to numerous metabolic genes, including those associated with mitochondrial biogenesis, and regulates the oxidative metabolism cooperatively with alternative NF-kB [50].

Kraft, C. S. et al. (2006) have shown that increased signals of myogenesis (MyoD, Myf5) are associated with a metabolic transition from glycolysis to oxidative phosphorylation, with a consequent increase in mitochondrial biogenesis [51]. These assumptions are not only in line with our data in the HC-CR groups but remarkably with the MFRs downregulations (vs HC-CR) and reduced expression of markers of mitochondrial biogenesis observed in the SD-CR-PM, that follow the same notion.

Another report, also in agreement with our data on MRFs expressions and AMPK observed in the HC-CRs, postulate that AMPK-α1 is the predominant AMP isoform expressed in satellite cells, and AMPK-α1 deficiency in satellite cells impairs their activation and myogenic differentiation during muscle regeneration [52].

Then we analyzed the AMPK-SIRT1-mTOR network, which is implicated in the regulation of many cellular and metabolic processes, autophagy, and cell homeostasis.

Cellular stresses due to low glucose and nutrient deprivation cause a rise in the AMP/ATP ratio, thus AMPK becomes activated (T183-T172 results). AMPK activity suppresses anabolic pathways like gluconeogenesis, glycogen, fatty acid, triglyceride, cholesterol, and protein synthesis (mTOR-p70SK-E2 pathway), and triggers catabolic pathways such as glycolysis, glucose uptake, and fatty acid oxidation to restore appropriate energy levels in the cell.

We observed in this work significantly increased transcript and protein expressions in the different AMPK subunits, and particularly in AMPK activity (T183-T172) in the HC-CR, in both PM and QF-*VM* (vs SD and SD-CR); results that were also associated with metabolic fiber transition towards the oxidative phenotype, and elevated mRNA/protein expressions of PGC-1α. These findings follow the notion that activation of AMPK leads to mitochondrial biogenesis by regulating PGC-1α which in turn improves the ATP synthesis capacity of the cell and promotes gene transcription in mitochondria, in skeletal muscles in response to chronic energy deprivation [53]. Moreover, Jager et al. [11] have also demonstrated that AMPK’s energy control function is exerted by activating mitochondrial biogenesis. This effect is realized by direct phosphorylation of PPAR-γ coactivator 1α (PGC-1α), the master regulator of this process, and while AMPK promotes the synthesis of new mitochondria, it also influences their turnover by activating their degradation through the autophagic process (mitophagy) [10].

Similar but not the same as our experimental design, Gutiérrez-Casado, et al. (2019) have shown that in skeletal muscle from CR mice, dietary fat influences mitochondrial mass and ultrastructure and may play a role in processes such as auto- and mitophagy and mitochondrial dynamics during aging [54].

Although we did not analyze mitophagy, the above-reported data may suggest that our animals previously fed HCD before CR (HC-CR), due to increased AMPK activity, may have elevated mitophagy activity (vs SD and SD-CR), and further studies in the levels of autophagy and senescence would add valuable insights in the understanding of how HCD background affects CR.

An important question that remains to be acknowledged here is why animals fed standard food, subjected to CR (SD-CR), presented lower AMPK activity (vs SD and HC-CR) in the two skeletal muscles?.

To answer this, we must take into consideration that the mechanisms of activation and the dynamics of AMPK are complex phenomena, not yet fully understood, and although it is recognized that phosphorylation at Threonine 172 (AMPK α2) - Threonine 183 (AMPK α1) is involved in AMPK activation, it may also be regulated by several other upstream kinase and pathways including CaMKK, Sirt1, LKB, CD36, ROS, allosteric regulation by AMP, AMP/ATP ratio, and the folliculin interacting protein 1 (Fnip1), among others [55–58]. Thus, we cannot rule out that distinct upstream regulators, stress signaling pathways, etc., are underpinning the decrease in AMPK activity in the SD-CR.

Interestingly, AMPK activity has been implicated in lipid accumulation in skeletal muscle cells through FTO-dependent demethylation of N6-methyladenosine [59]. Another report proposed that AMPK regulates muscle lipid storage, and reducing AMPK kinase activity decreases muscle perilipin-3 expression (which is consistent with our data) and intramyocellular triglyceride content [60].

Based upon the relation between AMPK with lipid storage/metabolism, our data in the SD-CR (in line with the HC and HC-CR data) appear to be linked at least in part, with energy status (demand/supply) and contents of INTM/IM lipid infiltration.

Moreover, changes in the levels of various hormones have been revealed to activate or inhibit AMPK [61–64], suggesting that its regulation and physiological relevance are far more complex than what we have covered in this manuscript.

SIRT1, which values (ng/ml) were slightly increased, but not statistically significant, in the CR-treated groups could be rationalized as prolonged exposures to CR in rodents have shown to have stronger effects on SIRT1 in several different tissues [8]. Nevertheless, we emphasize here that the regulatory properties of SIRT1 may, nonetheless, be remarkably more intricate, as SIRT1 activity relies also on intracellular levels of nicotinamide [65], post-translational modifications [66], and/or interactions with several other protein-mediators [67, 68].

mTOR activity, assayed in this work by the expression of mTOR-S2448, was reduced in the CR-treated groups (SD-CR-QF-*VM* was not statistically significant; vs SD); results that were expected as it is well documented that CR negatively regulates the mammalian target of rapamycin. However, considering that the mTOR pathways respond to various signals, including the nutrients (glucose and branched-chain amino acids), energy (ATP/AMP ratio), hypoxia, and growth factors (IGF-1/AKT pathway), etc., and that our data on T183-T172 (AMPK activity) showed mRNA and protein downregulations in the animals feed standard food previous to CR (SD-CR), another question mark related to which upstream mechanisms, other than AMPK activation, are implicated in mTOR inhibition, warrants deeper investigation.

It is now widely recognized that the adipose tissue is an essential and highly active metabolic-endocrine organ that secretes peptide hormones including leptin, cytokines (especially TNFα), adipsin and acylation-stimulating protein (ASP), angiotensinogen, plasminogen activator inhibitor-1 (PAI-1), adiponectin, resistin, etc., as well as steroids hormones. [69–71].

In this work we choose to investigate adiponectin, not only because of its numerous anti-aging properties but because the characterization of the skeletal muscle tissues after HCD-CR demonstrated contrasting amounts of lipid infiltration in the groups.

Previous reports have inversely correlated adiponectin circulating levels with obesity [72, 73], and positively associated it with CR [74, 75]. Curiously, Miller et, al. (2017), although they found a significant inverse relationship between HMW adiponectin and fat mass in mice of 10 or 20 months of age, no changes at 30 months of age were evidenced, indicating that in lean animals, the levels of HMW adiponectin, but not total adiponectin, are related to adiposity and that this relationship is sensitive to age. [20]. Moreover, Amany A Saleh, et al. (2020) found that adiponectin serum levels increase mainly due to secretion by the central adipose tissue but it does not reflect the tissue-specific expression of adiponectin and receptors, and this may vary in the different organs [76].

Our data showed that CR did not increase tissue-specific adiponectin protein expressions in the animals previously fed standard food (SD-CR-PM and -QF-*VM*), but it significantly upregulated adiponectin in the animals previously fed HCD (HC-CR-PM and -QF-*VM*). Because adiponectin is mainly produced by the adipocytes, we would expect lower protein expression of tissue-specific adiponectin in the SD-CR groups (SD-CR displayed markedly reduced lipid content). However, no changes were observed, compared to those found in the SD. This might be attributed to the fact that mechanistically, adiponectin, as a protective response against metabolic insults, can also be produced by the myocytes, *in vitro* and *in vivo* [77–80].

Surprisingly, the HC-PM displayed elevated translational expression of adiponectin (vs SD). Of note, HC-PM showed elevated amounts of fat infiltration and marked plasticity (fiber transition to the glycolytic phenotype), which was consistent with a report demonstrating that intramyocellular adiponectin levels are primarily associated with type IIA/D fibers containing elevated intramyocellular lipid accumulation [78].

It is evident that the magnitude of the subject embraced in this work goes far beyond the scope of the collected data. Thus, we would like to acknowledge this matter as one of the limitations that we have encountered so far. Here we merely present evidence of how HCD alters genetic patterns in the skeletal muscles of animals subjected to CR with the same BMI and how HCD in combination with CR affects specific key anti-aging mechanisms boosted by CR alone. That being said, further mechanistic studies on this topic are urgently required.

Also, it is important to mention that protein post-transcriptionally mRNA regulation or post-translational modifications (methylation, acetylation), differential protein degradation (proteasome-mediated or autophagy-mediated protein breakdown), pre-existing lysine acyl modifications, including succinylation, malonylation, glutarylation, crotonylation, beta-hydroxyisobutyrylation, 3-hydroxy 3-methylglutaryl, and fatty acylation, etc. [81, 82], influence protein structure and function, thus the transcriptional and translational expressions, in some of the genes analyzed in this manuscript, would not be always in line with each other.

Another aspect of our work that needs to be considered is that the data reflects the result of specific experimental conditions like animal species, age, tissue-specific expression, and characteristics of the dietary interventions, among others, so whether the results could be or could be not replicated, relies on the particularities of the experimental setup.

## CONCLUSIONS

Herein we present empirical evidence showing significant interactions between HCD and CR. The skeletal muscles analyzed, in the experimental and control groups, displayed metabolic and physiologic heterogeneity and remarkable plasticity, according to the dietary interventions.

HCD in combination with CR not only altered genetic activation patterns of satellite SC markers but also boosted the expression of MRFs and key regulators of mitochondrial biogenesis, which in turn were also associated with metabolic fiber transition.

Our data prompt us to theorize that the effects of CR may vary according to the physiologic, metabolic, and genetic peculiarities of the skeletal muscle described here and that INTM/IM lipid infiltration and tissue-specific fuel-energy status (demand/supply) both hold dependent-interacting roles with other key anti-aging mechanisms triggered by CR.

Finally, in this manuscript, we expose an unexplored area from which controversy may arise regarding the effects of CR on skeletal muscles in higher organisms that have been subject to caloric overload.

Systematic integration of an HCD with CR appears to bring potential benefits for skeletal muscle function and energy metabolism. However, at this stage of our research, an optimal balance between the two dietary conditions, where anti-aging effects can be accomplished, is under intensive investigation in combination with other tissues with distinct metabolic demands, and organ structures at different levels of organization within the whole-body system.

## MATERIALS AND METHODS

### Food design

The animal food was developed by Beijing Keao Xieli Feed Co., Ltd (Beijing Chaoyang district, Yangshan road, number 4). The standard food (3.1 kcal/100 g) composition can be seen in the Supplementary Table 1. A hypercaloric food rich in fatty acids and carbohydrates (HCD; 5.5 kcal/100 g) was designed to induce obesity in the animal model (Supplementary Table 2).

### Generation of the animal model and dietary interventions

Female CD-1 (ICR) mice were maintained in a specific pathogen-free animal facility in individual and ventilated cages and were housed at 23° C under a 12-hour dark/light cycle. Water and food were given *ad libitum* prior to the dietary interventions*.* This study was approved by the Institutional Review Board of the Chengdu Jinjiang Maternity and Child Health Hospital and all methods were performed under the relevant guidelines and regulations.

For the generation of the animal models, a group (Figure 1) initially composed by ~ 12-month-old mice (n=80) fed *ad libitum* 3.1 kcal/100 g standard food (First stage) was divided (second stage) into a group fed *ad libitum* standard food (n=30) and another group of animals switched to a 5.5 kcal/100 g *ad libitum* HCD for a period of 17 weeks (n=50). Then, mice previously fed standard food that continued with the same diet (n=10) became the standard diet control group (SD). Animals previously fed standard food, subjected to CR for a period of 13 weeks with a diet consisting of a 60% of the *ad libitum* 3.1 kcal/100 g calorie intake (n=19), became the standard diet-calorie restriction group (SD-CR); Animals fed HCD diet for a period of 17 weeks that continued with the same nourishment (n=14) became the high-calorie control group (HC); Animals fed HCD for a period of 17 weeks and then switched to CR (3.1 kcal/100 g food equivalent to 60% of the 5.5 kcal/100 g *ad libitum* calorie intake) for a period of 13 weeks (n=33) became the high calorie-calorie restriction group (HC-CR). The daily amount of food of the CR-restricted groups was adjusted according to decreasing body weight.

Mice were monitored daily by laboratory members and by animal health technicians. Before the experimental endpoint, the mice experienced minimal pain and stress during routine handling, bodyweight determination, and blood collection from the tail vein for measurement of blood glucose levels.

No ill or deceased mice were observed before the experimental endpoint (when the mice were 19 to 20 months old). Animals were euthanized, under the same metabolic conditions, by the cervical dislocation technique.

### Tissue collection

Muscle tissues were collected from the quadriceps femoris *vastus medialis* (QF-*VM*) of the right lower limb and the right pectoralis major (PM) of the chest. The samples were flash-frozen in liquid nitrogen and stored until the execution of the experiments.

### Oil red O staining assay for frozen sections

PM and QF-*VM* frozen tissues from the SD (n=6), SD-CR (n=8), HCD (n=9), and HCD-CR (n=12) were cut into 8μm thick sections with a cryostat (Leica CM1860 UV cryomicrotome, Leica Biosystems) and mounted on glass slides, then air-dried for 30-60min at room temperature and fixed in ice-cold 10% formalin for 5-10 minutes. The step was repeated twice and the slides were immediately rinsed with distilled water. After drying for several minutes, the slides were placed in absolute propylene glycol for 2-5 minutes and stained in pre-warmed oil red O solution for 8-10 minutes. Samples were treated with 85% propylene glycol solution for 2-5 minutes and rinsed with 2 changes of distilled water. Slides were finally stained in Mayer’s hematoxylin for 30 minutes, placed in distilled water, and then mounted with an aqueous mounting medium for microscope visualization. Area distribution of frozen section tissues was calculated with the ImageJ software.

### Succinic dehydrogenase assay for the determination of metabolic muscle fiber type

Metabolic fiber type (oxidative and glycolytic) in PM and QF-*VM* from the SD (n=6), SD-CR (n=8), HCD (n=9), and HCD-CR (n=12) was determined with the succinate dehydrogenase (SDH) staining solution (Tetra-salt method; Batch # G2000) kit (Beijing Solarbio Science and Technology Co., Ltd.). Six or more transverse sections (12 mm thick) were obtained with a cryostat (Leica CM1860 UV cryomicrotome, Leica Biosystems). Briefly, the flash-frozen sections were immersed in NBT incubation solution for 20 min at 37 C. Samples were rinsed with distilled water, and coverslips were mounted onto glass slides with a natural balsam for microscopic visualization. Myofibers were processed with a digital slide scanner (MoticEasyScan, Motic Microscopy) linked to a Motic digital slide assistant image capture software. Areas were randomly selected for the determination of metabolic fiber type.

### Quantitative real-time polymerase chain reaction (qPCR) assay

Tissues preserved in liquid nitrogen were homogenized, and the RNA was extracted with Takara RNAiso PLUS Total RNA Extraction Reagent (Takara Bio, Inc.). DNA was extracted with a TIANamp Genomic DNA Kit (TIANGEN). The total nucleic acid concentration and optical density (OD) were assayed by UV spectrophotometry. cDNA was obtained with a Takara kit (RR047Q). qPCR was performed with 100 ng of target DNA; the expression of the pair box gene (PAX) 3, PAX7, PAX9, cluster of differentiation 34 (CD34), myoblast determination protein 1 (MyoD), myogenic factor 5 (Myf5), adipose-type cytoplasmic fatty acid binding protein (Fabp4), perilipin, Insulin growth factor 1 (IGF-1), IGF-1 receptor (IGF-1R), nitric oxide synthase (eNOS), peroxisome proliferator-activated receptor-gamma coactivator-1alpha (PGC-1α, mitochondrial import receptor subunit TOM20 homolog (TOMM20), translocase of outer mitochondrial membrane 40 homolog (TOMM40), mitochondrial import inner membrane translocase subunit Tim9 (TIMM9), NADH dehydrogenase [ubiquinone] iron-sulfur protein 3, mitochondrial (NDUFS3), ATP synthase, H+ transporting, mitochondrial F1 complex, gamma polypeptide 1 (ATP5C1), AMP-activated protein kinase (AMPK) Alpha 1-2, Beta 1-2, Gamma 1-2, AMPK phosphorylated at Threonine 183 and 172 (T183-T172), mammalian target of rapamycin (mTOR), and 18s ribosomal RNA (18s rRNA) or GAPDH (the two reference genes) was evaluated through relative quantification with SYBR Premix Ex Taq™ (Takara RR420Q) using an ABI7500 instrument (*Applied* Biosystems, Foster City, CA). The samples were run in triplicate and the list of primers can be seen in Supplementary Table 3.

### Western blot analysis

The tissues were homogenated with 5 volumes of Radioimmunoprecipitation Assay (RIPA) buffer (Solarbio^®^ life sciences), and the supernatants were fractionated by SDS-PAGE. The proteins were quantified with BCA protein quantification kit (Solarbio^®^ life sciences), transferred to PVDF fiber membranes (Bio-RAD), and blocked with 5 % blocking solution for Western blot (Roche). The membranes were then exposed to Anti-Fabp4 antibody [EPR3579] ab92501(Abcam; dilution 1/2000); Anti-Perilipin-1 [EPR3753(2)] ab172907 (Abcam; dilution: 1/2000); IGF1 Rabbit mAb #A0830 (ABclonal; dilution: 1/500); Anti-IGF1 Receptor antibody [EPR19322] ab182408 (Abcam; dilution: 1/1000); Anti-eNOS antibody [EPR19296] ab199956 (Abcam; dilution: 1/1000); PGC1 alpha Rabbit pab, #A11971 (ABclonal; dilution: 1/1000); Anti-AMPK alpha 1 + AMPK alpha 2 antibody [EPR19549] ab207442 (Abcam; dilution: 1/1000); Anti-AMPK beta 1 antibody [Y367] ab32112 (Abcam; dilution: 1/2500); Recombinant Anti-AMPK gamma 1 antibody [y308] ab32508 (Abcam; dilution: 1/5000); Recombinant Anti-AMPK alpha 1 (phospho T183) + AMPK alpha 2 (phospho T172) antibody [EPR5683] ab133448 (Abcam; dilution: 1/5000); Anti-mTOR antibody [EPR390(N)] ab134903 (Abcam; dilution: 1/5000); Anti-mTOR (phosphor s2448) antibody [EPR426(2)] ab109268 (Abcam; dilution: 1/5000); Anti-Adiponectin antibody [EPR17019] ab181281 (Abcam; dilution: 1/1000); Anti-GAPDH antibody [EPR 16891] – Loading Control ab181602 (Abcam; dilution: 1/5000). Immunodetection was performed using Goat Anti-Rabbit IgG H&L (HRP) ab6721 (Abcam; dilution: 1/3000) secondary antibody, and an enhanced chemiluminescence device (Bio-Rad gel imager).

### Mitochondrial DNA copy number assay

DNA of PM and QF-*VM* tissues from the SD (n=9), SD-CR (n=16), HCD (n=14), and HCD-CR (n=30) was isolated with the TIANamp Genomic DNA Kit (TIANGEN) and the mitochondrial DNA was analyzed with a Mouse Mitochondrial DNA Copy Number Assay Kit (Detroit R&D, Inc.) through comparison of mitochondrial (mt) and nuclear (n) DNA measured by qPCR.

The mitochondrial DNA copy number was calculated as follows:

ΔCt1 = Ct (control mitochondrial DNA) − Ct (control nuclear DNA).

ΔCt2 = Ct (experimental group mitochondrial DNA) − Ct (experimental group nuclear DNA).

The Mouse Mitochondrial DNA Copy Number Assay Kit protocol is annexed as Supplementary Material 1.

### Telomerase detection assay

Tissue homogenates were analyzed with a TRAPeze RT Telomerase Detection Kit (Millipore) for fluorometric detection and real-time quantification of telomerase activity.

Briefly, 50 to 100 mg of PM and QF-*VM* frozen tissues from the SD (n=10), SD-CR (n=19), HC (n=14), and HC-CR (n=33) were homogenized and resuspended in 200 μl of CHAPS Lysis Buffer. The samples were incubated in ice for 30 minutes and then centrifuged at 12,000 x g for 20 minutes at 4C. One hundred sixty μl of the supernatant was transferred into a fresh tube and used to determine protein concentration. The remaining extract was used to perform the RT telomerase assay according to the kit protocol (Supplementary Material 2).

### NAD-dependent deacetylase sirtuin-1 assay

One hundred mg of PM and QF-*VM* tissue homogenates from the SD (n=10), SD-CR (n=19), HC (n=14), and HC-CR (n=33) were analyzed with a Mouse NAD-Dependent Deacetylase Sirtuin-1 (SIRT1) ELISA Kit (CUSABIO) for the quantitative determination of SIRT1, according to its manual instructions (Supplementary Material 3).

### Statistical analysis

The data were analyzed with a two-tailed unpaired t-test with Welch’s correction for comparisons of two groups or a two-way ANOVA with a Bonferroni posthoc analysis to determine interactions between multiple groups. Correlations were tested by Pearson analysis and the data were processed using GraphPad Prism 8.3.0. Western blot analysis and protein densitometry were conducted with Image Lab 6.1. A P value < 0.05 was considered to indicate statistical significance.

## Supplementary Material

Supplementary Table 1

Supplementary Table 2

Supplementary Table 3

Supplementary Table 4

Supplementary Material 1

Supplementary Material 2

Supplementary Material 3

## References

[1] McCay CM, Crowell MF, Maynard LA. The effect of retarded growth upon the length of life span and upon the ultimate body size. 1935. Nutrition. 1989; 5:155–71. 2520283

[2] Cerletti M, Jang YC, Finley LW, Haigis MC, Wagers AJ. Short-term calorie restriction enhances skeletal muscle stem cell function. Cell Stem Cell. 2012; 10:515–19. 10.1016/j.stem.2012.04.002 22560075 PMC3561899

[3] Seale P, Sabourin LA, Girgis-Gabardo A, Mansouri A, Gruss P, Rudnicki MA. Pax7 is required for the specification of myogenic satellite cells. Cell. 2000; 102:777–86. 10.1016/s0092-8674(00)00066-0 11030621

[4] Buckingham M, Bajard L, Chang T, Daubas P, Hadchouel J, Meilhac S, Montarras D, Rocancourt D, Relaix F. The formation of skeletal muscle: from somite to limb. J Anat. 2003; 202:59–68. 10.1046/j.1469-7580.2003.00139.x 12587921 PMC1571050

[5] Cornelison DD, Wold BJ. Single-cell analysis of regulatory gene expression in quiescent and activated mouse skeletal muscle satellite cells. Dev Biol. 1997; 191:270–83. 10.1006/dbio.1997.8721 9398440

[6] Zhang K, Sha J, Harter ML. Activation of Cdc6 by MyoD is associated with the expansion of quiescent myogenic satellite cells. J Cell Biol. 2010; 188:39–48. 10.1083/jcb.200904144 20048262 PMC2812847

[7] Beauchamp JR, Heslop L, Yu DS, Tajbakhsh S, Kelly RG, Wernig A, Buckingham ME, Partridge TA, Zammit PS. Expression of CD34 and Myf5 defines the majority of quiescent adult skeletal muscle satellite cells. J Cell Biol. 2000; 151:1221–34. 10.1083/jcb.151.6.1221 11121437 PMC2190588

[8] Nisoli E, Tonello C, Cardile A, Cozzi V, Bracale R, Tedesco L, Falcone S, Valerio A, Cantoni O, Clementi E, Moncada S, Carruba MO. Calorie restriction promotes mitochondrial biogenesis by inducing the expression of eNOS. Science. 2005; 310:314–17. 10.1126/science.1117728 16224023

[9] Cohen HY, Miller C, Bitterman KJ, Wall NR, Hekking B, Kessler B, Howitz KT, Gorospe M, de Cabo R, Sinclair DA. Calorie restriction promotes mammalian cell survival by inducing the SIRT1 deacetylase. Science. 2004; 305:390–92. 10.1126/science.1099196 15205477

[10] Cetrullo S, D’Adamo S, Tantini B, Borzi RM, Flamigni F. mTOR, AMPK, and Sirt1: Key Players in Metabolic Stress Management. Crit Rev Eukaryot Gene Expr. 2015; 25:59–75. 10.1615/critreveukaryotgeneexpr.2015012975 25955819

[11] Jäger S, Handschin C, St-Pierre J, Spiegelman BM. AMP-activated protein kinase (AMPK) action in skeletal muscle via direct phosphorylation of PGC-1alpha. Proc Natl Acad Sci USA. 2007; 104:12017–22. 10.1073/pnas.0705070104 17609368 PMC1924552

[12] Iwabu M, Yamauchi T, Okada-Iwabu M, Sato K, Nakagawa T, Funata M, Yamaguchi M, Namiki S, Nakayama R, Tabata M, Ogata H, Kubota N, Takamoto I, et al. Adiponectin and AdipoR1 regulate PGC-1alpha and mitochondria by Ca^2+^ and AMPK/SIRT1. Nature. 2010; 464:1313–19. 10.1038/nature08991 20357764

[13] Lambert AJ, Wang B, Yardley J, Edwards J, Merry BJ. The effect of aging and caloric restriction on mitochondrial protein density and oxygen consumption. Exp Gerontol. 2004; 39:289–95. 10.1016/j.exger.2003.12.009 15036388

[14] Faitg J, Leduc-Gaudet JP, Reynaud O, Ferland G, Gaudreau P, Gouspillou G. Effects of Aging and Caloric Restriction on Fiber Type Composition, Mitochondrial Morphology and Dynamics in Rat Oxidative and Glycolytic Muscles. Front Physiol. 2019; 10:420. 10.3389/fphys.2019.00420 31114501 PMC6503296

[15] Redman LM, Smith SR, Burton JH, Martin CK, Il’yasova D, Ravussin E. Metabolic Slowing and Reduced Oxidative Damage with Sustained Caloric Restriction Support the Rate of Living and Oxidative Damage Theories of Aging. Cell Metab. 2018; 27:805–15.e4. 10.1016/j.cmet.2018.02.019 29576535 PMC5886711

[16] Coluzzi E, Leone S, Sgura A. Oxidative Stress Induces Telomere Dysfunction and Senescence by Replication Fork Arrest. Cells. 2019; 8:19. 10.3390/cells8010019 30609792 PMC6356380

[17] Whittemore K, Vera E, Martínez-Nevado E, Sanpera C, Blasco MA. Telomere shortening rate predicts species life span. Proc Natl Acad Sci USA. 2019; 116:15122–27. 10.1073/pnas.1902452116 31285335 PMC6660761

[18] Vera E, Bernardes de Jesus B, Foronda M, Flores JM, Blasco MA. Telomerase reverse transcriptase synergizes with calorie restriction to increase health span and extend mouse longevity. PLoS One. 2013; 8:e53760. 10.1371/journal.pone.0053760 23349740 PMC3551964

[19] Yamauchi T, Kadowaki T. Adiponectin receptor as a key player in healthy longevity and obesity-related diseases. Cell Metab. 2013; 17:185–96. 10.1016/j.cmet.2013.01.001 23352188

[20] Miller KN, Burhans MS, Clark JP, Howell PR, Polewski MA, DeMuth TM, Eliceiri KW, Lindstrom MJ, Ntambi JM, Anderson RM. Aging and caloric restriction impact adipose tissue, adiponectin, and circulating lipids. Aging Cell. 2017; 16:497–507. 10.1111/acel.12575 28156058 PMC5418198

[21] Kaikaus RM, Bass NM, Ockner RK. Functions of fatty acid binding proteins. Experientia. 1990; 46:617–30. 10.1007/BF01939701 2193826

[22] Miyoshi H, Souza SC, Endo M, Sawada T, Perfield JW 2nd, Shimizu C, Stancheva Z, Nagai S, Strissel KJ, Yoshioka N, Obin MS, Koike T, Greenberg AS. Perilipin overexpression in mice protects against diet-induced obesity. J Lipid Res. 2010; 51:975–82. 10.1194/jlr.M002352 19797618 PMC2853465

[23] Hausman GJ, Basu U, Du M, Fernyhough-Culver M, Dodson MV. Intermuscular and intramuscular adipose tissues: Bad vs. good adipose tissues. Adipocyte. 2014; 3:242–55. 10.4161/adip.28546 26317048 PMC4550684

[24] Morton RW, Sato K, Gallaugher MP, Oikawa SY, McNicholas PD, Fujita S, Phillips SM. Muscle Androgen Receptor Content but Not Systemic Hormones Is Associated With Resistance Training-Induced Skeletal Muscle Hypertrophy in Healthy, Young Men. Front Physiol. 2018; 9:1373. 10.3389/fphys.2018.01373 30356739 PMC6189473

[25] Tengan CH, Rodrigues GS, Godinho RO. Nitric oxide in skeletal muscle: role on mitochondrial biogenesis and function. Int J Mol Sci. 2012; 13:17160–84. 10.3390/ijms131217160 23242154 PMC3546744

[26] Li PA, Hou X, Hao S. Mitochondrial biogenesis in neurodegeneration. J Neurosci Res. 2017; 95:2025–29. 10.1002/jnr.24042 28301064

[27] Austin S, St-Pierre J. PGC1α and mitochondrial metabolism--emerging concepts and relevance in ageing and neurodegenerative disorders. J Cell Sci. 2012; 125:4963–71. 10.1242/jcs.113662 23277535

[28] Weng G, Zhou B, Liu T, Huang Z, Yang H. Sitagliptin promotes mitochondrial biogenesis in human SH-SY5Y cells by increasing the expression of PGC-1α/NRF1/TFAM. IUBMB Life. 2019; 71:1515–21. 10.1002/iub.2076 31290617

[29] García-Prieto CF, Gil-Ortega M, Plaza A, Manzano-Lista FJ, González-Blázquez R, Alcalá M, Rodríguez-Rodríguez P, Viana M, Aránguez I, Gollasch M, Somoza B, Fernández-Alfonso MS. Caloric restriction induces H_2_O_2_ formation as a trigger of AMPK-eNOS-NO pathway in obese rats: Role for CAMKII. Free Radic Biol Med. 2019; 139:35–45. 10.1016/j.freeradbiomed.2019.05.016 31100477

[30] Inoki K, Kim J, Guan KL. AMPK and mTOR in cellular energy homeostasis and drug targets. Annu Rev Pharmacol Toxicol. 2012; 52:381–400. 10.1146/annurev-pharmtox-010611-134537 22017684

[31] Ghosh HS, McBurney M, Robbins PD. SIRT1 negatively regulates the mammalian target of rapamycin. PLoS One. 2010; 5:e9199. 10.1371/journal.pone.0009199 20169165 PMC2821410

[32] Bartolomé A, García-Aguilar A, Asahara SI, Kido Y, Guillén C, Pajvani UB, Benito M. MTORC1 Regulates both General Autophagy and Mitophagy Induction after Oxidative Phosphorylation Uncoupling. Mol Cell Biol. 2017; 37:e00441–17. 10.1128/MCB.00441-17 28894028 PMC5686580

[33] Tanner CJ, Barakat HA, Dohm GL, Pories WJ, MacDonald KG, Cunningham PR, Swanson MS, Houmard JA. Muscle fiber type is associated with obesity and weight loss. Am J Physiol Endocrinol Metab. 2002; 282:E1191–96. 10.1152/ajpendo.00416.2001 12006347

[34] Hickey MS, Carey JO, Azevedo JL, Houmard JA, Pories WJ, Israel RG, Dohm GL. Skeletal muscle fiber composition is related to adiposity and *in vitro* glucose transport rate in humans. Am J Physiol. 1995; 268:E453–57. 10.1152/ajpendo.1995.268.3.E453 7900793

[35] Lillioja S, Young AA, Culter CL, Ivy JL, Abbott WG, Zawadzki JK, Yki-Järvinen H, Christin L, Secomb TW, Bogardus C. Skeletal muscle capillary density and fiber type are possible determinants of *in vivo* insulin resistance in man. J Clin Invest. 1987; 80:415–24. 10.1172/JCI113088 3301899 PMC442253

[36] Wade AJ, Marbut MM, Round JM. Muscle fibre type and aetiology of obesity. Lancet. 1990; 335:805–08. 10.1016/0140-6736(90)90933-v 1969558

[37] Zhang L, Zhou Y, Wu W, Hou L, Chen H, Zuo B, Xiong Y, Yang J. Skeletal Muscle-Specific Overexpression of PGC-1α Induces Fiber-Type Conversion through Enhanced Mitochondrial Respiration and Fatty Acid Oxidation in Mice and Pigs. Int J Biol Sci. 2017; 13:1152–62. 10.7150/ijbs.20132 29104506 PMC5666330

[38] Pette D, Staron RS. Transitions of muscle fiber phenotypic profiles. Histochem Cell Biol. 2001; 115:359–72. 10.1007/s004180100268 11449884

[39] Zierath JR, Hawley JA. Skeletal muscle fiber type: influence on contractile and metabolic properties. PLoS Biol. 2004; 2:e348. 10.1371/journal.pbio.0020348 15486583 PMC521732

[40] Timmers S, Konings E, Bilet L, Houtkooper RH, van de Weijer T, Goossens GH, Hoeks J, van der Krieken S, Ryu D, Kersten S, Moonen-Kornips E, Hesselink MK, Kunz I, et al. Calorie restriction-like effects of 30 days of resveratrol supplementation on energy metabolism and metabolic profile in obese humans. Cell Metab. 2011; 14:612–22. 10.1016/j.cmet.2011.10.002 22055504 PMC3880862

[41] Chung JH, Manganiello V, Dyck JR. Resveratrol as a calorie restriction mimetic: therapeutic implications. Trends Cell Biol. 2012; 22:546–54. 10.1016/j.tcb.2012.07.004 22885100 PMC3462230

[42] Lam YY, Peterson CM, Ravussin E. Resveratrol vs. calorie restriction: data from rodents to humans. Exp Gerontol. 2013; 48:1018–24. 10.1016/j.exger.2013.04.005 23624181

[43] Dunn SE, Kari FW, French J, Leininger JR, Travlos G, Wilson R, Barrett JC. Dietary restriction reduces insulin-like growth factor I levels, which modulates apoptosis, cell proliferation, and tumor progression in p53-deficient mice. Cancer Res. 1997; 57:4667–72. 9354418

[44] Sonntag WE, Lynch CD, Cefalu WT, Ingram RL, Bennett SA, Thornton PL, Khan AS. Pleiotropic effects of growth hormone and insulin-like growth factor (IGF)-1 on biological aging: inferences from moderate caloric-restricted animals. J Gerontol A Biol Sci Med Sci. 1999; 54:B521–38. 10.1093/gerona/54.12.b521 10647962

[45] Fontana L, Klein S. Aging, adiposity, and calorie restriction. JAMA. 2007; 297:986–94. 10.1001/jama.297.9.986 17341713

[46] Poudel SB, Dixit M, Neginskaya M, Nagaraj K, Pavlov E, Werner H, Yakar S. Effects of GH/IGF on the Aging Mitochondria. Cells. 2020; 9:1384. 10.3390/cells9061384 32498386 PMC7349719

[47] Boldrin L, Ross JA, Whitmore C, Doreste B, Beaver C, Eddaoudi A, Pearce DJ, Morgan JE. The effect of calorie restriction on mouse skeletal muscle is sex, strain and time-dependent. Sci Rep. 2017; 7:5160. 10.1038/s41598-017-04896-y 28698572 PMC5505993

[48] Al-Kafaji G, Golbahar J. High glucose-induced oxidative stress increases the copy number of mitochondrial DNA in human mesangial cells. Biomed Res Int. 2013; 2013:754946. 10.1155/2013/754946 23984405 PMC3745925

[49] Komatsu H. Myosin ATPase. In: xPharm: The Comprehensive Pharmacology Reference. 2007.

[50] Shintaku J, Peterson JM, Talbert EE, Gu JM, Ladner KJ, Williams DR, Mousavi K, Wang R, Sartorelli V, Guttridge DC. MyoD Regulates Skeletal Muscle Oxidative Metabolism Cooperatively with Alternative NF-κB. Cell Rep. 2016; 17:514–26. 10.1016/j.celrep.2016.09.010 27705798 PMC5059110

[51] Kraft CS, LeMoine CM, Lyons CN, Michaud D, Mueller CR, Moyes CD. Control of mitochondrial biogenesis during myogenesis. Am J Physiol Cell Physiol. 2006; 290:C1119–27. 10.1152/ajpcell.00463.2005 16531567

[52] Fu X, Zhu MJ, Dodson MV, Du M. AMP-activated protein kinase stimulates Warburg-like glycolysis and activation of satellite cells during muscle regeneration. J Biol Chem. 2015; 290:26445–56. 10.1074/jbc.M115.665232 26370082 PMC4646303

[53] Zong H, Ren JM, Young LH, Pypaert M, Mu J, Birnbaum MJ, Shulman GI. AMP kinase is required for mitochondrial biogenesis in skeletal muscle in response to chronic energy deprivation. Proc Natl Acad Sci USA. 2002; 99:15983–87. 10.1073/pnas.252625599 12444247 PMC138551

[54] Gutiérrez-Casado E, Khraiwesh H, López-Domínguez JA, Montero-Guisado J, López-Lluch G, Navas P, de Cabo R, Ramsey JJ, González-Reyes JA, Villalba JM. The Impact of Aging, Calorie Restriction and Dietary Fat on Autophagy Markers and Mitochondrial Ultrastructure and Dynamics in Mouse Skeletal Muscle. J Gerontol A Biol Sci Med Sci. 2019; 74:760–69. 10.1093/gerona/gly161 30010806 PMC6521922

[55] Carling D. The AMP-activated protein kinase cascade--a unifying system for energy control. Trends Biochem Sci. 2004; 29:18–24. 10.1016/j.tibs.2003.11.005 14729328

[56] Samovski D, Sun J, Pietka T, Gross RW, Eckel RH, Su X, Stahl PD, Abumrad NA. Regulation of AMPK activation by CD36 links fatty acid uptake to β-oxidation. Diabetes. 2015; 64:353–59. 10.2337/db14-0582 25157091 PMC4303974

[57] Hardie DG. AMP-activated protein kinase as a drug target. Annu Rev Pharmacol Toxicol. 2007; 47:185–210. 10.1146/annurev.pharmtox.47.120505.105304 16879084

[58] Xiao L, Liu J, Sun Z, Yin Y, Mao Y, Xu D, Liu L, Xu Z, Guo Q, Ding C, Sun W, Yang L, Zhou Z, et al. AMPK-dependent and -independent coordination of mitochondrial function and muscle fiber type by FNIP1. PLoS Genet. 2021; 17:e1009488. 10.1371/journal.pgen.1009488 33780446 PMC8031738

[59] Wu W, Feng J, Jiang D, Zhou X, Jiang Q, Cai M, Wang X, Shan T, Wang Y. AMPK regulates lipid accumulation in skeletal muscle cells through FTO-dependent demethylation of N^6^-methyladenosine. Sci Rep. 2017; 7:41606. 10.1038/srep41606 28176824 PMC5296945

[60] Kleinert M, Parker BL, Chaudhuri R, Fazakerley DJ, Serup A, Thomas KC, Krycer JR, Sylow L, Fritzen AM, Hoffman NJ, Jeppesen J, Schjerling P, Ruegg MA, et al. mTORC2 and AMPK differentially regulate muscle triglyceride content via Perilipin 3. Mol Metab. 2016; 5:646–55. 10.1016/j.molmet.2016.06.007 27656402 PMC5021677

[61] Lim CT, Kola B, Korbonits M. AMPK as a mediator of hormonal signalling. J Mol Endocrinol. 2010; 44:87–97. 10.1677/JME-09-0063 19625456

[62] Bayliss JA, Lemus MB, Stark R, Santos VV, Thompson A, Rees DJ, Galic S, Elsworth JD, Kemp BE, Davies JS, Andrews ZB. Ghrelin-AMPK Signaling Mediates the Neuroprotective Effects of Calorie Restriction in Parkinson’s Disease. J Neurosci. 2016; 36:3049–63. 10.1523/JNEUROSCI.4373-15.2016 26961958 PMC4783502

[63] Park SH, Ryu SY, Yu WJ, Han YE, Ji YS, Oh K, Sohn JW, Lim A, Jeon JP, Lee H, Lee KH, Lee SH, Berggren PO, et al. Leptin promotes K(ATP) channel trafficking by AMPK signaling in pancreatic β-cells. Proc Natl Acad Sci U S A. 2013; 110:12673–8. 10.1073/pnas.1216351110 23858470 PMC3732963

[64] Ruderman NB, Carling D, Prentki M, Cacicedo JM. AMPK, insulin resistance, and the metabolic syndrome. J Clin Invest. 2013; 123:2764–72. 10.1172/JCI67227 23863634 PMC3696539

[65] Anderson RM, Bitterman KJ, Wood JG, Medvedik O, Cohen H, Lin SS, Manchester JK, Gordon JI, Sinclair DA. Manipulation of a nuclear NAD^+^ salvage pathway delays aging without altering steady-state NAD+ levels. J Biol Chem. 2002; 277:18881–90. 10.1074/jbc.M111773200 11884393

[66] Yang Y, Fu W, Chen J, Olashaw N, Zhang X, Nicosia SV, Bhalla K, Bai W. SIRT1 sumoylation regulates its deacetylase activity and cellular response to genotoxic stress. Nat Cell Biol. 2007; 9:1253–62. 10.1038/ncb1645 17934453 PMC3201724

[67] Kim JE, Chen J, Lou Z. DBC1 is a negative regulator of SIRT1. Nature. 2008; 451:583–86. 10.1038/nature06500 18235501

[68] Zhao W, Kruse JP, Tang Y, Jung SY, Qin J, Gu W. Negative regulation of the deacetylase SIRT1 by DBC1. Nature. 2008; 451:587–90. 10.1038/nature06515 18235502 PMC2866287

[69] Galic S, Oakhill JS, Steinberg GR. Adipose tissue as an endocrine organ. Mol Cell Endocrinol. 2010; 316:129–39. 10.1016/j.mce.2009.08.018 19723556

[70] McGown C, Birerdinc A, Younossi ZM. Adipose tissue as an endocrine organ. Clin Liver Dis. 2014; 18:41–58. 10.1016/j.cld.2013.09.012 24274864

[71] Guerre-Millo M. Adipose tissue hormones. J Endocrinol Invest. 2002; 25:855–61. 10.1007/BF03344048 12508947

[72] Arita Y, Kihara S, Ouchi N, Takahashi M, Maeda K, Miyagawa J, Hotta K, Shimomura I, Nakamura T, Miyaoka K, Kuriyama H, Nishida M, Yamashita S, et al. Paradoxical decrease of an adipose-specific protein, adiponectin, in obesity. Biochem Biophys Res Commun. 1999; 257:79–83. 10.1006/bbrc.1999.0255 10092513

[73] Turer AT, Khera A, Ayers CR, Turer CB, Grundy SM, Vega GL, Scherer PE. Adipose tissue mass and location affect circulating adiponectin levels. Diabetologia. 2011; 54:2515–24. 10.1007/s00125-011-2252-z 21779869 PMC4090928

[74] Zhu M, Miura J, Lu LX, Bernier M, DeCabo R, Lane MA, Roth GS, Ingram DK. Circulating adiponectin levels increase in rats on caloric restriction: the potential for insulin sensitization. Exp Gerontol. 2004; 39:1049–59. 10.1016/j.exger.2004.03.024 15236764

[75] Combs TP, Berg AH, Rajala MW, Klebanov S, Iyengar P, Jimenez-Chillaron JC, Patti ME, Klein SL, Weinstein RS, Scherer PE. Sexual differentiation, pregnancy, calorie restriction, and aging affect the adipocyte-specific secretory protein adiponectin. Diabetes. 2003; 52:268–76. 10.2337/diabetes.52.2.268 12540596

[76] Saleh AA, Tayel SI, Shalaby AG, El Naidany SS. Role of Adiponectin Gene and Receptor Polymorphisms and Their mRNA Levels with Serum Adiponectin Level in Myocardial Infarction. Appl Clin Genet. 2020; 13:241–52. 10.2147/TACG.S282843 33376382 PMC7755379

[77] Goto A, Ohno Y, Ikuta A, Suzuki M, Ohira T, Egawa T, Sugiura T, Yoshioka T, Ohira Y, Goto K. Up-regulation of adiponectin expression in antigravitational soleus muscle in response to unloading followed by reloading, and functional overloading in mice. PLoS One. 2013; 8:e81929. 10.1371/journal.pone.0081929 24324732 PMC3855747

[78] Krause MP, Liu Y, Vu V, Chan L, Xu A, Riddell MC, Sweeney G, Hawke TJ. Adiponectin is expressed by skeletal muscle fibers and influences muscle phenotype and function. Am J Physiol Cell Physiol. 2008; 295:C203–12. 10.1152/ajpcell.00030.2008 18463233 PMC2493546

[79] Liu Y, Chewchuk S, Lavigne C, Brûlé S, Pilon G, Houde V, Xu A, Marette A, Sweeney G. Functional significance of skeletal muscle adiponectin production, changes in animal models of obesity and diabetes, and regulation by rosiglitazone treatment. Am J Physiol Endocrinol Metab. 2009; 297:E657–64. 10.1152/ajpendo.00186.2009 19531641

[80] Delaigle AM, Jonas JC, Bauche IB, Cornu O, Brichard SM. Induction of adiponectin in skeletal muscle by inflammatory cytokines: *in vivo* and *in vitro* studies. Endocrinology. 2004; 145:5589–97. 10.1210/en.2004-0503 15319349

[81] Audagnotto M, Dal Peraro M. Protein post-translational modifications: In silico prediction tools and molecular modeling. Comput Struct Biotechnol J. 2017; 15:307–19. 10.1016/j.csbj.2017.03.004 28458782 PMC5397102

[82] Sabari BR, Zhang D, Allis CD, Zhao Y. Metabolic regulation of gene expression through histone acylations. Nat Rev Mol Cell Biol. 2017; 18:90–101. 10.1038/nrm.2016.140 27924077 PMC5320945

