# Supplementary material for: The consequences of a high-calorie diet background before calorie restriction on skeletal muscles in a mouse model
Source: Aging (Albany NY). 2021 Jun 24;13(12):16834–58. doi: 10.18632/aging.203237 (PMC8266348; doi:10.18632/aging.203237)
Supplement: Supplementary Table 1 [file aging-13-203237-s001.pdf]

SUPPLEMENTARY TABLE

Supplementary Table 1. Standard food composition.

Number	Inspection item	Test result (%)	Test method
1	Crude protein	19.7	GB/T6432-1994 7.2
2	Coarse fiber	3.0	GB/T6434-2006
3	Coarse ash	5.94	GB/T6438-2007
4	Calcium	1.17	GB/T6436-2018
5	Total phosphorus	0.89	GB/T6437-2002
6	Crude fat	5.3	GB/T6433-2006 9.3
7	Water	9.5	GB/T6435-2014 8.1

Supplied by the manufacturer company: Beijing Keao Xieli Feed Co., Ltd. (Beijing Chaoyang district, Yangshan road, number 4).
*The standard food provides 3.1 kcal/kg of energy.
