# Supplementary material for: The consequences of a high-calorie diet background before calorie restriction on skeletal muscles in a mouse model
Source: Aging (Albany NY). 2021 Jun 24;13(12):16834–58. doi: 10.18632/aging.203237 (PMC8266348; doi:10.18632/aging.203237)
Supplement: Supplementary Table 3 [file aging-13-203237-s003.pdf]

Supplementary Table 3. List of primers.

Oligo name	Sequence forward (5' to 3')	Sequence reverse (5' to 3')	Product size (bp)	Accession number[a]
18s rRNA	GTAACCCTTTGAACCCCATT	CCATCCAATCGGTAGTAGCG		
Adiponectin	AAGGACAAGGCCGTTCTCT	TATGGGTAGTTGCAGTCAGTTGG	219	NM_009605.5
Adipo R1	TTCTTCCTCATGGCTGTGATGT	AAGAAGCGCTCAGGAATTCG	71	XM_021197931.2
AdipoR2	TGCAGCCATTATAGTCTCCCAG	GAATGATTCCACTCAGGCCTAG	101	XM_021190928.2
AMPKα1	GTCGACGTAGCTCCAAGACC	ATCGTTTTCCAGTCCCTGTG	232	XM_011245321.3
AMPKα1	CGCCTCTAGTCCTCCATCAG	ATGTCACACGCTTTGCTCTG	219	XM_029539581.1
AMPKβ2	GTTGCTGTTGCTTGTTCCAA	ATACTGTGCCTGCCTCTGCT	149	XM_021163526.2
AMPKβ2	ACCCAAGCACAGCTCTAGACACAA	AGGGTAGTTCCTTGCCTCACACAT	119	XM_006500841.2
AMPKγ1	TCCCTAGACCTCACCACACC	GTCTGCACAGCACAAGAACC	191	XM_021183007.2
AMPKγ1	ATTGACCCTATCAGTGGGAACGCA	TCCGATTCCAAGCTCATCCAGGTT	132	XM_030254045.1
ATP5C1	CATGGACAACGCCAGCAAGA	TTTACCTCTTGTCTGAGGATGCAAC	165	NM_001112738.1
CD34	GTTATTTCCTGATGAACCGTCG	CTCCACCATTCTCCGTGTAATA	84	XM_021198209.1
eNOS	AAGATTGCCTCGGTTTGTTG	AAGATTGCCTCGGTTTGTTG	89	XM_006535639.4
Fabp4	ATGATCATCAGCGTAAATGG	GCCTTTCATAACACATTCCA	246	XM_021157748.2
GAPDH	AGGTCGGTGTGAACGGATTTG	GGGGTCGTTGATGGCAACA	95	XM_017321385.2
IGF-1	TGGATGCTCTTCAGTTCGTG	GTCTTGGGCATGTCAGTGTG	220	XM_030244912.2
IGF-1R	GTGGGGGCTCGTGTTTCTC	GATCACCGTGCAGTTTTCCA	127	XM_006540643.5
mTOR	TTCAATCCATAGCCCCGTCT	CAAAGAGCTGCATCACTCGT	150	XM_006539077.3
Myf5	CTGTCTGGTCCCGAAAGAAC	AAGCAATCCAAGCTGGACAC	103	XM_006513319.2
MyoD	CCCCGGCGGCAGAATGGCTACG	GGTCTGGGTTCCCTGTTCTGTGT	234	NM_010866.2
NDUFS3	GCTTCGAGGGACATCCTTTC	AGTTACTTGGTTTCAGGCTTCT	223	XM_021152446.2
PAX3	TCGGAGGAGTATTTATCAACGG	CAATTTTCTTCTCCACGTCAGG	231	XM_021156775.1
PAX7	GTCATGAGCATCCTTAGCAAC	CCATACTGGCTGTACTGGTAG	242	XM_021160851.1
PAX9	GAAACACATCCGGACTTACAAG	GCTGCTTGTATGAGTCGTAATG	185	XM_021179351.1
Perilipin	CTGTGTGCAATGCCTATGAGA	CTGGAGGGTATTGAAGAGCCG	171	XM_011250776.3
PGC1-α	TATGGAGTGACATAGAGTGTGCT	CCACTTCAATCCACCCAGAAAG	134	XM_036164894.1
T-cadherin	CATCGAAGCTCAAGATATGG	GATTTCCATTGATGATGGTG	230	XM_021169677.2
TIMM9	AATATGGCTGCACAGATACC	TTCAGGTTTCACCTCTCTTG	135	XM_017315088.2
TOMM20	GAAACAGAAGCTTGCTAAGGAG	GTCACCTTGTGCTAGTAACTCT	127	XM_021170680.2
TOMM40	TGAACAGTAACTGGATCGTG	GGAGGACATCAAGTCTTTCC	390	XM_030242769.1
