# Supplementary material for: The consequences of a high-calorie diet background before calorie restriction on skeletal muscles in a mouse model
Source: Aging (Albany NY). 2021 Jun 24;13(12):16834–58. doi: 10.18632/aging.203237 (PMC8266348; doi:10.18632/aging.203237)

Mouse Mitochondrial DNA Copy Number Assay Kit

(44 reactions)

Catalog Number: MCN 3
Store at -20°C.
FOR RESEARCH USE ONLY

Introduction: This DNA analysis kit is for the determination of mouse mitochondrial DNA copy number, *in vivo* and *in vitro*, by the comparison of mitochondrial (mt) and nuclear (n) DNA measured by real-time PCR.

Kit Contents:

- 96 well PCR plate
- rtPCR reaction mix.
- Validated primers (10 µM) to quantify mitochondrial DNA (mtDNA).
- Validated primers (10 µM) to quantify nuclear DNA (nDNA).
- Positive control [2.5 ng/ µl] (isolated total DNA from liver of B6 mouse).

Not Included in Kit:

- DNA isolation Kit
- Nuclease-free water
- PCR Tubes and Caps

Thermal cycler program:

- Preprogram PCR machine for this profile:
  a. 95°C, 10 min
     (40 Cycles)
  b. 95°C, 15 sec
  c. 60°C, 60 sec

Real time PCR procedure: The following procedure is for each 20 µL reaction. Increase all amounts proportionally according to the total number of tubes.

- Per PCR tube (20 µL *Rx*), mix the following:
  a. 1 µL forward primer
  b. 1 µL reverse primer
  c. 8 µL sample contain genomic DNA/ 8 µL of positive control
  d. 10 µL rtPCR reaction mix

Recommended concentration: Between 0.3 to 5.0 ng/µL

How to Calculate Mitochondrial Copy Number:

ΔCt1 = Ct (mitochondria-control) – Ct (nucleus-control)
ΔCt2 = Ct (mitochondrial-experimental) – Ct (nucleus-experimental)

$\Delta\Delta Ct$ = Sample ΔCt – average ΔCt control
mtDNA fold change = $2^{-\Delta\Delta Ct}$

Total DNA isolated from mouse liver

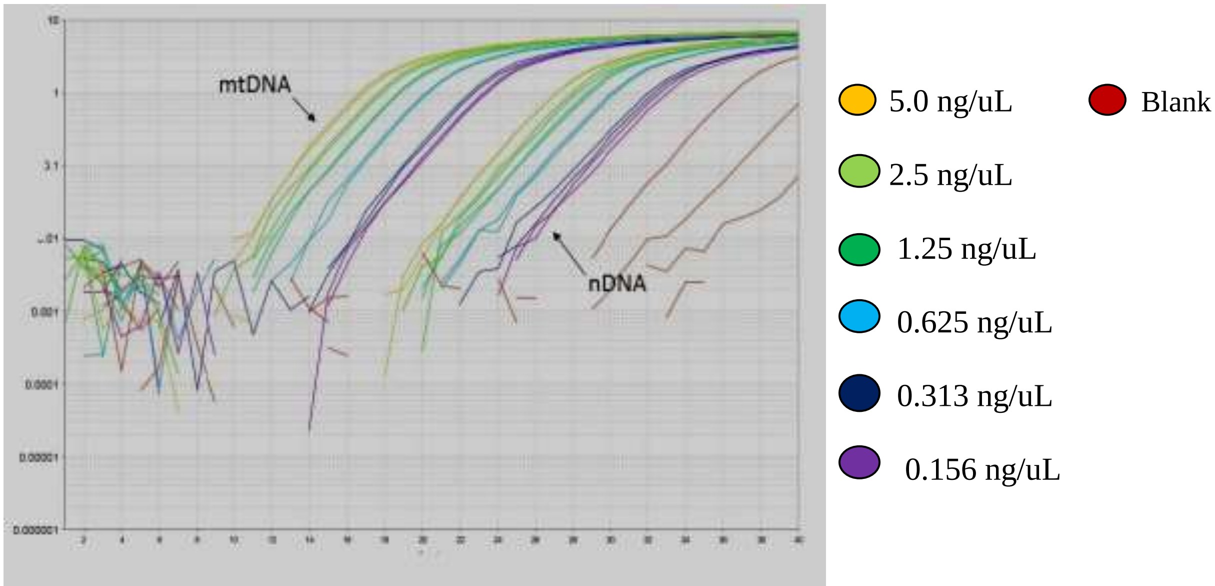

Suggested assay plate layout: n = nucleus; mt = mitochondria; BLK = blank

	1	2	3	4	5	6	7	8	9	10	11	12
A	nBLK	nS3	nS7	nS11	nS15	nS19	mtBLK	mtS3	mtS7	mtS11	mtS15	mtS19
B	nBLK	nS3	nS7	nS11	nS15	nS19	mtBLK	mtS3	mtS7	mtS11	mtS15	mtS19
C	nPC	nS4	nS8	nS12	nS16	nS20	mtPC	mtS4	mtS8	mtS12	mtS16	mtS20
D	nPC	nS4	nS8	nS12	nS16	nS20	mtPC	mtS4	mtS8	mtS12	mtS16	mtS20
E	nS1	nS5	nS9	nS13	nS17	nS21	mtS1	mtS5	mtS9	mtS13	mtS17	mtS21
F	nS1	nS5	nS9	nS13	nS17	nS21	mtS1	mtS5	mtS9	mtS13	mtS17	mtS21
G	nS2	nS6	nS10	nS14	nS18	nS22	mtS2	mtS6	mtS10	mtS14	mtS18	mtS22
H	nS2	nS6	nS10	nS14	nS18	nS22	mtS2	mtS6	mtS10	mtS14	mtS18	mtS22

272

Plot of C_T versus DNA concentration

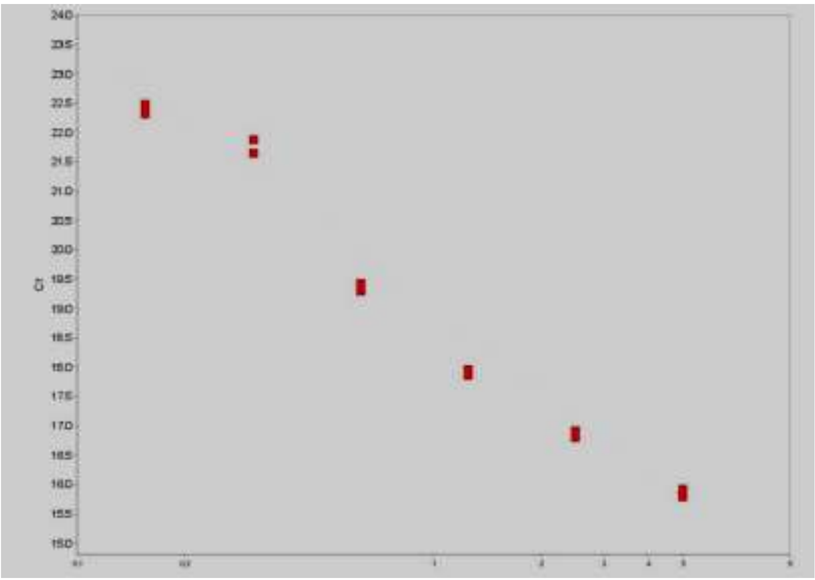

References

1- Wein et al., Oncology Letters; 6: 1098-1102, 2013
2- Santos et al., Free Radical Biology & Medicine; 51: 1849–1860, 2011
3- Santos et al., Invest Ophthalmol Vis Sci 2011 Nov 11; 52 (12): 8791-8798
4- Edwards et al,. Diabetologia; 53: 160–169, 2010.

Ref 3- Santos et al

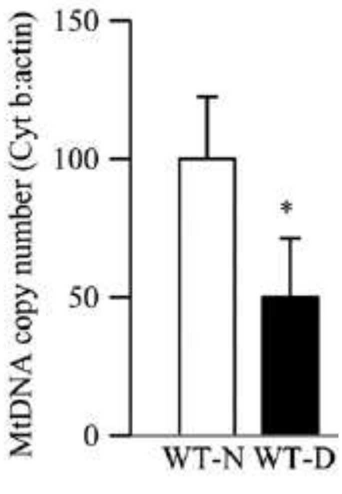

Ref 4- Edwards et al.

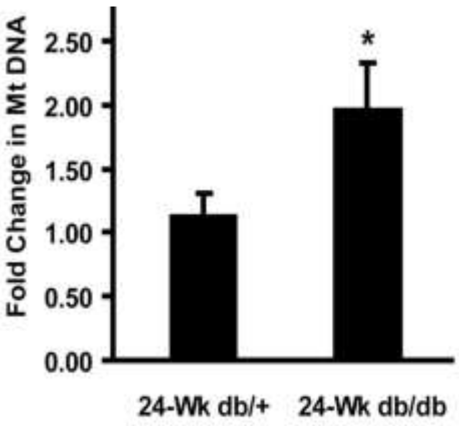

Supplement: Supplementary Material 1 [file aging-13-203237-s005.pdf]
