# Supplementary material for: The consequences of a high-calorie diet background before calorie restriction on skeletal muscles in a mouse model
Source: Aging (Albany NY). 2021 Jun 24;13(12):16834–58. doi: 10.18632/aging.203237 (PMC8266348; doi:10.18632/aging.203237)
Supplement: Supplementary Material 2 [file aging-13-203237-s006.pdf]

TRAPeze® Kit RT Telomerase Detection Kit

Catalog No. S7710

For Research Use Only
Not for use in diagnostic procedures

USA & Canada Phone: +1(800) 645-5476
In Europe, please contact Customer Service:
France: 0825.045.645;
Spain: 901.516.645 Option 1
Germany: 01805.045.645
Italy: 848.845.645
United Kingdom: 0870.900.46.45
For other locations across the world please visit www.millipore.com/offices

Table of Contents

I. Introduction 1
Using This Manual 1
Background 1
Principles of the Technique 2
The Amplifluor® Primer System 2
TRAPeze® RT Telomerase Detection Kit with Amplifluor® Primers 3
Fig. 1: TRAPeze® RT Telomerase Detection Kit Scheme 3

II. Kit Components 4
Table 1: TRAPeze® RT Telomerase Detection Kit 4
Materials Required But Not Supplied 4
Equipment and Supplies 4
Reagents 4
Precautions 4

III. Protocol 5
Fig. 2: TRAPeze® RT Telomerase Detection Kit Flow Chart 5
Extract Preparation 5
Table 2: Sample Concentration and Quantity for Assay 6
Experimental Design 6
Controls 7
Table 3: Dilution of Control Template TSR8 7
Assay Design 9
TRAPeze® RT Telomerase Detection Kit 10
Assay Setup 10
Required Controls and Samples 10
Optional Amplification Control and Samples 10
PCR Amplification 11
Real-time Fluorescence Data 11
Fig. 3 Serial Dilutions of TSR8 Quantitation Control 11
Data Analysis 12
Table 4: Data for the Generation of the TSR8 Standard Curves 12
Logarithmic Plot 12
Fig. 4 Logarithmic Plot of the TSR8 Standard Curve 13
Sensitivity of the Assay 13

IV. Troubleshooting 13

V. Appendix 15
Laboratory Setup and Precautions 16
Fig. 5: TRAPeze® RT Telomerase Detection Kit Station Setup 16
TRAPeze® RT Telomerase Detection Kit Station Setup (Area 1) 17
Excitation and Emission Filters 17

VI. References 18
References Cited in the Manual 18
Disclaimers 20
Warranty 20

Introduction

Using this Manual

This manual accommodates both the novice and the experienced TRAPeze® Telomerase Detection Kit (Cat. No. S7700, S7707 and S7750) user. These protocols are presented in a streamlined manner. However, users are directed to sections that provide supplemental information by notations in the protocol. The novice user is advised to read the entire manual prior to using the TRAPeze® RT Kit, particularly Sec. III. *Protocols*, *Experimental Design*. Directions for preparing some of the required reagents can be found in Sec. V. *Appendix*.

For Research Use Only; Not for use in diagnostic procedures

Background

Telomeres are specific structures found at the end of chromosomes in eukaryotes. In human chromosomes, the telomeres consist of thousands of copies of 6 base repeats (TTAGGG)[1-3]. It has been suggested that telomeres protect chromosome ends since damaged chromosomes lacking telomeres undergo fusion, rearrangement and translocation [2]. In somatic cells, telomere length is progressively shortened with each cell division both *in vivo* and *in vitro* [4-7] due to the inability of the DNA polymerase complex to synthesize the very 5' end of the lagging strand [8,9].

Telomerase is a ribonucleoprotein that synthesizes and directs the telomeric repeats onto the 3' end of existing telomeres using its RNA component as a template [10-14]. Telomerase activity has been shown to be specifically expressed in immortal cells, cancer and germ cells [15,16] where it compensates for telomere shortening during DNA replication and thus stabilizes telomere length [7,17]. These observations have led to a hypothesis that telomere length may function as a “mitotic clock” to sense the number of cell divisions and eventually signal replicative senescence or programmed cell death when a critical telomere length is achieved. Therefore, expression of telomerase activity in cancer cells may be a necessary and essential step for tumor development and progression (16,18-20). The causal relationship between expression of telomerase and telomere length stabilization and the extension of the life span of the human cell has recently been reported [21].
The development of a sensitive and efficient PCR-based telomerase activity detection method, TRAP (Telomeric Repeat Amplification Protocol) [15, 22], has made possible large scale surveys of telomerase activity in human cells and tissues [15, 23-29]. To date, telomerase activity has been detected in over 85% of all tumors tested spanning more than 20 different types of cancers [30-31].

The TRAPeze® RT Telomerase Detection Kit is a highly sensitive in vitro assay for the fluorometric detection and real time quantification of telomerase activity. It incorporates refinements to the original TRAP assay that were first introduced in the gel-based TRAPeze® Telomerase Detection Kit (Cat. No. S7700) and adds the ability to quantitate telomerase activity using fluorescence energy transfer (ET) primers similar to the TRAPEeze® XL Telomerase Detection Kit (Cat. No. S7707). As in the original TRAPeze® Kit, primer sequence modifications that reduce amplification artifacts and PCR controls for standard curve generation are included. In addition, both the TRAPeze® RT and XL Kits use fluorescence energy transfer (ET) primers to generate fluorescently labeled TRAP products which permit nonisotopic, quantitative analysis of telomerase activity.

The unique design of these ET primers (Amplifluor® primers) allows detection and quantification of telomerase activity by directly measuring real time fluorescence emission in the reaction vessels. Since Amplifluor® primers will fluoresce only upon incorporation into the TRAP products, post–PCR sample manipulations such as electrophoretic gel or ELISA analyses are eliminated, thereby reducing the the risk of carry-over contamination. Quantitative analysis is not compromised when detection is performed in a high-throughput 96-well format unlike platforms utilizing a qualitative ELISA. Additionaly, an additional stand alone control is provided separately to assess PCR inhibitors that may be present in experimental samples.

Principles of the Technique

The Amplifluor® Primer System

The fluorometric detection of telomerase activity by the TRAPeze® RT Telomerase Detection Kit is accomplished through the use of Amplifluor® primers. This patented technology (33) is based on the concept of energy transfer (ET), a process whereby energy shifted from an excited fluorophore to an acceptor moiety results in quenching of the fluorescence emission. In order for this quenching to occur, the donor and acceptor molecules must be in close physical proximity to each other. Amplifluor® primers, which contain energy transfer moieties, are designed to emit a fluorescence signal only when they are incorporated into PCR products. Therefore, the net increase of fluorescence in the reaction vessel directly correlates to the amount of amplified DNA produced in the reaction.

Amplifluor® primers consist of a 3' end sequence complementary to the target sequence and a 5' end hairpin structure. The fluorophore (energy donor) and the quencher DABSYL (4-(dimethylamine)azo benzene sulfonic acid) are in close proximity within the 5' hairpin (Figure 1, line B). As the primer is incorporated into a double-stranded PCR product, the hairpin is unfolded through the activity of the polymerase (Figure 1, line C). In this extended conformation, the distance between the fluorophore and quencher is increased and a fluorescence signal is generated (Figure 1, line D).

TRAPeze® RT Telomerase Detection Kit with Amplifluor® Primers

The TRAPeze® RT Telomerase Detection Kit is a one buffer, two enzyme system utilizing polymerase chain reaction (PCR) and Amplifluor® primers (34). In the first step of the reaction (Figure 1, line A), the telomerase enzyme adds a number of telomeric repeats (GGTTAG) onto the 3' end of a substrate oligonucleotide (TS). In the next steps (Figure 1, line B and C), the extended products are amplified by the second enzyme, Taq Polymerase, using PCR with the TS and fluorescein-labeled Amplifluor® RP (reverse) primers. This generates a fluorescent ladder of products with 6 base increments starting at 61 nucleotides: 61, 67, 73, 79, etc. The fluorescence emission produced is directly proportional to the amount of TRAP products generated (Figure 1, line D).

Figure 1: TRAPeze® RT Telomerase Detection Kit Scheme

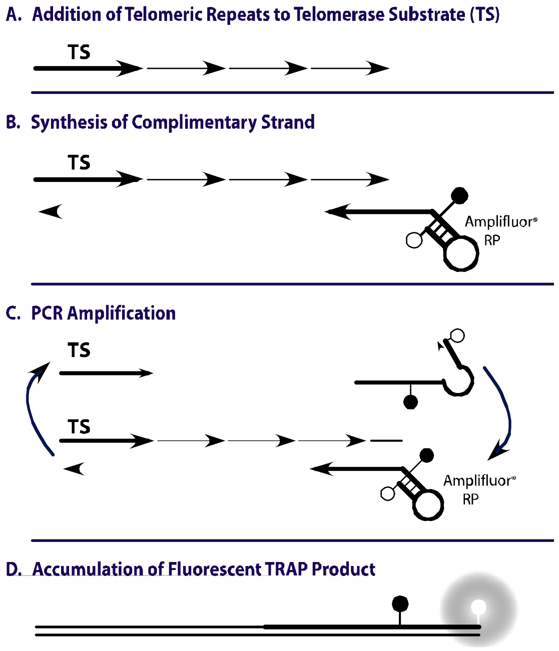

Each short arrow on line A symbolizes a 6 nucleotide-long telomeric repeat (GGTTAG). The small filled circles represent DABSYL while the open circles represent the fluorophore quenched by DABSYL. The large circle on line D depicts unquenched fluorescence emission.

In addition to the 5X TRAPeze® RT Reaction Mix, a second 5X TRAPeze® RT Control Reaction Mix including a TSK control template is provided as an artificial PCR control to assess PCR inhibitor status and as a positive control for PCR amplification. The control template can also be used to normalize signal obtained from the 5X TRAPeze® RT Reaction Mix for normalized quantitation of telomerase activity similar to the assay conditions in the TRAPeze® RT Kit (see Sec. III. *Protocol, Experimental Design*).

The TRAPeze® RT Telomerase Detection Kit is intended for use with real time PCR instrument detection. Standard curves using the included TSR8 quantitation control provide a method for quantitation of telomerase activity relative to TSR8 product amplification. (see Sec. III. *Protocol; Data Analysis*).

Kit Components

The kit provides enough reagents to perform 224 TRAPeze® RT reactions. With these reagents, 74 experimental samples and control samples can be analyzed in triplicate (for details of the experimental design, see Sec. III. *Protocol, Experimental Design*).

**TRAPeze® RT Telomerase Detection Kit Contents**		
**S7710 FR (-20°C)**		
**Component**	**Item #**	**Quantity**
**CHAPS Lysis Buffer**	90436	13.5 mL
**5X TRAPeze® RT Reaction Mix**	2003668	1.12 mL
**5X TRAPeze® Control Reaction Mix**	2003670	1.12 mL
**PCR - Grade Water (Store at 2°C to 8°C)**	90411	8.2 mL
**TSR8*: (quantitation control template)**	CS213229	45 µL
**TSK*: (pcr inhibition/normalization control)**	2003665	45 µL
**S7710 DFR (-80°C)**		
**Component**	**Storage Conditions**	**Quantity**
**Control Cell Pellet: Telomerase positive cells**	-75°C to -85°C	106 cells

** Caution – refer to Sec. II.* Kit Components, Precautions.

Materials Required But Not Supplied

Reagents

- Antibody-mediated, hot start Taq polymerase
  Note: Titanium™ Taq (BD Clontech®) is recommended for use with the TRAPeze® RT Telomerase Detection System. Other antibody hot start enzymes may be suitable for use with this assay with optimization. Non-hot start and chemically modified hot start enzymes are not recommended for use with Amplifluor®. Contact Chemicon Technical Service at (800) 437-7500 or techserv@chemicon.com for more detailed information regarding enzyme selection.
- PBS (Mg^{2+}- and Ca^{2+}-free)
- Reagents for protein concentration measurement
- RNase inhibitor (for extract preparation from tissues)
- Bovuminar® Bovine Serum Albumin

Equipment and Supplies

- Real time fluorescence capable thermocycler with FAM detection (i.e. ABI Prism®7500/7700/7900, iCycler®, MJ Opticon™, Stratagene MX® 4000, etc.). When using iCycler®, use a blank well sample plate instead of the experimental plate for well factoring prior initiating telomerase extension step at 30°C. Product has not been tested on the ABI 7500 Fast system and assay may require optimization for performance on this instrument. (See Sec. V. *Appendix, Excitation and Emission Filters*)
- Optically clear 96-well plates and caps for real-time PCR amplification and detection
- If analyzing tissues, homogenization equipment as described in Sec. III. Protocol, *Extract Preparation*
- Aerosol resistant pipette tips (RNase-free)

III. Protocol

Precautions

Because the TRAPeze® RT Telomerase Detection Kit detects the activity of telomerase, a RNase sensitive ribonucleoprotein, and not merely the presence of the RNA or protein components of telomerase, the assay requires enzymatically active cell or tissue samples. Furthermore, due to the sensitivity of the TRAPeze® RT Kit, which can detect telomerase activity in a very small number of cells, a special laboratory setup and significant precautions are required to prevent contamination. Standard precautions used for preventing cross contamination on a PCR assay should be followed. It is strongly recommended that a designated set of pipettes be used exclusively for pipetting TSR8 control template. These pipettes should not be used for any other part of the assay.

1. TSR8 control template
2. PCR carry-over
3. RNase

These precautions are discussed in detail in **Sec. V. *Appendix, Laboratory Setup and Precautions* and *TRAPeze® RT Telomerase Detection Kit Station Setup* (Area 1).**

For Research Use Only. Not for use in diagnostic procedures.

Figure 2: TRAPeze® RT Telomerase Detection Kit Flow Chart

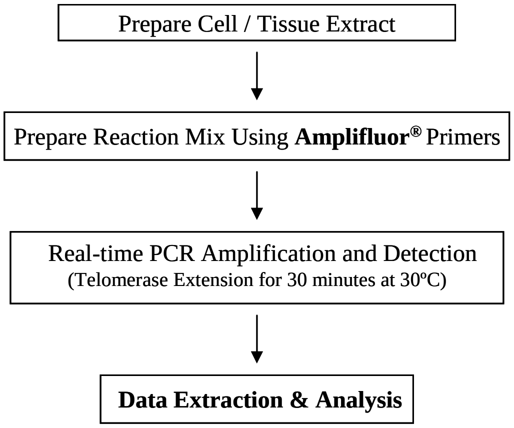

Extract Preparation

Note: *The volume of CHAPS Lysis Buffer used is adjusted for the number of cells to be extracted. To determine the volume of CHAPS Lysis Buffer for each sample, establish the cell number by counting or extrapolation from tissue weight.*

When preparing extracts from tumor samples, add RNase inhibitor to the CHAPS Lysis Buffer prior to the extraction for a final concentration of 100-200 units/mL.

1. Pellet the cells or tissue, wash once with PBS, repellet, and carefully remove all PBS. After removal of PBS, the cells or tissue pellet can be stored at -85°C to -75°C or kept on dry ice. Telomerase in frozen cells or tissues is stable for at least 1 year at -85°C to -75°C. When thawed for extraction, the cells or tissue should be resuspended immediately in CHAPS Lysis Buffer (step 2).

2a. Cells

Resuspend the cell pellet in 200 µL of CHAPS Lysis Buffer/10^5-10^6 cells. (Also use 200 µL of CHAPS Lysis Buffer for the preparation of the positive control cell extract in the kit.) Proceed to Step 3.

2b. Tissues

Prepare the extract according to one of the methods described below. Use 200 µL of CHAPS Lysis Buffer/40-100 mg of tissue.

Soft Tissues - Homogenization with Motorized Disposable Pestle: Mince the tissue sample with a sterile blade until a smooth consistency is obtained. Transfer the sample to a sterile 1.5 mL microcentrifuge tube, and add CHAPS Lysis Buffer. Keep the sample on ice and homogenize with a motorized pestle (~10 seconds) until a uniform consistency is achieved.

Connective Tissues - Freezing and Grinding: Place the tissue sample in a sterile mortar and freeze by adding liquid nitrogen. Pulverize the sample by grinding with a matching pestle. Transfer the thawed sample to a sterile 1.5 mL microcentrifuge tube, and resuspend it in an appropriate amount of CHAPS Lysis Buffer.

Connective Tissues - Mechanical Homogenizer: Mix the tissue sample with an appropriate volume of CHAPS Lysis Buffer in a sterile 1.5 mL microcentrifuge tube placed on ice. Homogenize with a mechanical homogenizer until a uniform consistency is achieved (~5 seconds). It is critical to keep the sample on ice during homogenization to prevent heat accumulation.

3. Incubate the suspension on ice for 30 minutes.
4. Spin the sample in a microcentrifuge at 12,000 x g for 20 minutes at 4°C.
5. Transfer 160 µL of the supernatant into a fresh tube and determine the protein concentration.

Table 2: Sample Concentration and Quantity for Assay

Sample Type	Concentration	Quantity
Cell Extract	10-750 ng/µL	<1.5 µg per assay
Tissue Extract	10-500 ng/µL	<1.5 µg per assay

6. Aliquot and quick-freeze the remaining extract on dry ice*, and store at -85°C to -75°C. The extract is stable for at least 12 months when stored at -85°C to -75°C.

***Note:** *The extracts from the TRAPeze® RT Kit should be quick-frozen on dry ice after each use. Aliquots should not be freeze-thawed more than 10 times to avoid loss of telomerase activity. In addition, aliquoting reduces the risk of contamination.*

Experimental Design

For a valid analysis of the results, two factors need to be considered: (1) appropriate controls and (2) the amount of cell/tissue extract to be used in each reaction.

Controls

For each TRAPeze® RT Telomerase Assay:

1. Generation of TSR8 Standard Curve:

   Use a designated set of pipettes exclusively for pippetting TSR8 control template and make sure that these pipettes are not used in any other part of the assay.

   Perform the TRAPeze® RT Telomerase Detection Kit using dilutions of TSR8 (control template) instead of the sample extract to generate a standard curve. TSR8 is an oligonucleotide with a sequence identical to the TS primer extended with 8 telomeric repeats $AG(GGTTAG)_7$. This standard curve permits the calculation of the amount of TS primers with telomeric repeats extended by telomerase in a given extract.

   The stock TSR8 (control template) concentration provided within the kit is 20 amoles/µL. Prepare 1:10 serial dilutions of the stock concentration with CHAPS Lysis Buffer to obtain TSR8 concentrations of 2 amoles/µL, 0.2 amoles/µL and 0.02 amoles/µL. Perform the TRAPeze® RT Assay using 2 µL of each TSR8 dilution including the 20 amoles/µL stock concentration. If desired, further dilutions may be performed to extend the dynamic range of the assay, as long as results for more dilute samples are detected significantly earlier than NTC samples.

Table 3: Dilution of Control Template TSR8

TSR8 Concentration	Volume of TSR8 Stock	Volume of Diluent CHAPS Lysis Buffer
1. 20 amoles/µL	No dilution	-
2. 2 amoles/µL	2 µL of #1	18 µL
3. 0.2 amoles/µL	2 µL of #2	18 µL
4. 0.02 amoles/ µl	2 µL of #3	18 µL

2. Telomerase Positive Extract Control

   Make a telomerase-positive cell extract using 200 µL of CHAPS Lysis Buffer and the control cell pellet (10^6 cells) provided in the kit. Aliquot the lysate and store at -75°C to -85°C. Dilute the stock aliquots 1:10 with CHAPS Lysis Buffer before use and dispense 2 µL per TRAPeze® RT Assay (2 µL = 1000 cells). Run one positive control reaction for each set of TRAPeze® RT Telomerase Assays.

3. Minus Telomerase Control

   Perform a TRAPeze® RT Assay with 2 µL CHAPS Lysis Buffer substituted for the cell/tissue extract.

   Note: *The detection of fluorescein labeled products in the minus telomerase control reaction suggests either: 1) the presence of primer-dimer PCR artifacts due to suboptimal PCR conditions; 2) the presence of PCR contamination (amplified TRAPeze® RT products or TSR8) carried over from another assay; or 3) the contamination of an assay component with the telomerase positive cell extract. Results from the Minus Telomerase Control should be compared to the results from the No Template Control to determine if signal generated from this sample is due to contamination or inherent Amplifluor primer dimers. (see Sec. IV. Troubleshooting).*

4. No Template Control (NTC)

   Perform a TRAPeze® RT Assay with 2 µL of Nuclease Free Water substituted for the cell/tissue extract. Due to design of this assay, a certain level of primer dimer formation inevitably occurs with the Amplifluor primers in the absence of telomerase activity. This NTC occurs below the lowest level of detection of the assay and will not affect data analysis when sample activity is detected above this lowest limit (see Sec. III. *Protocol, Data Analysis*).

5. Generation of TSK Normalization Standard Curve (Optional)

   An optional feature of the TRAPeze® RT Assay is an additional stand alone control template and reaction mix that can be used to assess PCR performance and inhibitors in the cell or tissue extract samples. Many cell/tissue extracts contain inhibitors of Taq polymerase, and thus could give potentially false-negative results. To distinguish this from other possibilities, the 5X TRAPeze® RT Control Reaction Mix contains Amplifluor primers tailored for amplification of an artifical TSK template. These primers and template produce a detectable 83bp amplification product that serves as a control for amplification efficiency in each reaction and as a positive control for PCR amplification. (see Sec. III. *Protocol, Data Analysis*). In addition to analyzing an aliquot of each sample with a fixed concentration of TSK template, a standard curve in the absence of sample may be performed to assign a quantitative value to TSK amplification of each sample that can then be used to normalize the values obtained from the TSR8 standard curve.

Perform the TRAPeze® RT Assay using dilutions of the TSK (control template) and CHAPs Buffer instead of the sample extract to generate a standard curve similar to TSR8 (see above).

The stock TSK template concentration provided within the kit is 0.2 amoles/µL. Prepare 1:10 serial dilutions of the stock concentration with CHAPS Lysis Buffer to obtain TSK concentrations of 0.02 amoles/µL, 0.002 amoles/µL and 0.0002 amoles/µL. Perform the TRAPeze® RT Assay using 2 µL of each TSK dilution including the 0.20 amoles/µL stock concentration.

For each sample

1. Heat treated telomerase negative control. Telomerase is a heat-sensitive enzyme. As a negative control, every sample extract to be evaluated should also be tested for heat sensitivity using the 5X TRAPeze® RT Reaction Mix. Heat treat 10 µL of each sample by incubating at 85°C for 10 minutes prior to the TRAPeze® RT Telomerase Detection to inactivate telomerase.

2. PCR positive control to assess amplification and presence of PCR inhibitors (Optional). An 5X TRAPeze® Control Reaction Mix is provided that can be used either as a qualitative PCR control reaction, or as an internal control for normalization of PCR inhibitors using a standard curve.

Assay Design

The TRAPeze® RT Telomerase Detection Kit is designed for the successful analysis of 74 experimental samples and controls when run in triplicate. Supposing 8 experimental samples (n) are analyzed at a time.

A1 – D3: 4 TSR8 quantitation controls

E1 – E3: **1000 cell equivalents** of telomerase positive extract control

F1 – F3: Minus telomerase control

G1 – G3: No Template Control (NTC)

A4 – H9: 8 experimental samples and heat-treated controls

A10 – H12: 8 experimental samples with TSK template and Control Reaction Mix (Optional)

	1	2	3	4	5	6	7	8	9	10	11	12
A	TSR8 40 amoles			Sample 1			Sample 1—Heat Treated			Sample 1		
B	TSR8 4 amoles			Sample 2			Sample 2—Heat Treated			Sample 2		
C	TSR8 0.4 amoles			Sample 3			Sample 3—Heat Treated			Sample 3		
D	TSR8 0.04 amoles			Sample 4			Sample 4—Heat Treated			Sample 4		
E	Positive Extract (1000 cells)			Sample 5			Sample 5—Heat Treated			Sample 5		
F	Minus Telomerase Control			Sample 6			Sample 6—Heat Treated			Sample 6		
G	NTC			Sample 7			Sample 7—Heat Treated			Sample 7		
H				Sample 8			Sample 8—Heat Treated			Sample 8		
	5X TRAPEZE® RT Reaction Mix			5X TRAPEZE® RT Reaction Mix			5X TRAPEZE® RT Reaction Mix			5X TRAPEZE® Control Reaction Mix		

Assay Setup

To determine the total number of reactions to be run in the assay, refer to Sec. III. *Protocol, Experimental Design*. Typically, for analysis of n number of sample extracts.

It is essential that optically clear PCR plates and caps be used when the PCR reactions will be measured directly in the wells.

1. Prepare a "Master Mix" by mixing the reagents outlined below **except** for the extract. Thaw all reagents, mix well and store on ice.

Required Controls and Samples:

5X TRAPEZE® RT Reaction Mix*	5.0 µL
Taq Polymerase (5 units/µL)	0.4 µL (2 Units)
Nuclease Free Water	17.6 µL
Samples: (TSR8 dilutions, Positive extract control, Telomerase Negative, NTC, Experimental Samples +/- heat treatment)	2.0 µL
**TOTAL VOLUME**	**25.0 µL**

Note: *Experimental sample can be Cell Extract (10 - 750 ng/µL) or Tissue Extract (10 - 500 ng/µL)*

Optional Amplification Control and Samples:

5X TRAPEZE® RT Control Reaction Mix*	5.0 µL
Taq Polymerase (5 units/µL)	0.4 µL (2 Units)
TSK Template***	2.0 µL
Nuclease Free Water	15.6 µL
Samples***	2.0 µL
**TOTAL VOLUME**	**25.0 µL**

**Upon first use, make aliquots of 5X TRAPeze® RT Reaction Mixes, which can be freeze-thawed no more than 5 times.*

****For control samples,0.004 amols of TSK can be used in the presence of 2 µL experimental sample for a qualitative assessment of ampliflication. For quantitative normalization, also perform a dilution series of TSK similar to TSR8 (Table 3) in the absence of sample for standard curve generation.*

2. Aliquot 23.0 µL of the Mix containing Taq polymerase into a PCR Plate used for your real-time instrument.
3. Add 2 µL of experimental samples, heat-inactivated extracts or controls into the designated well:
   a. Sample extracts: add 2 µL to each of the wells.
   b. Heat-inactivated controls: incubate 10 µL of each sample extract at 85°C for 10 minutes. Add 2 µL into each of the heat-inactivation control wells.
   c. TSR8 quantitation controls: add 2 µL of each TSR8 dilution including the stock concentration.
   d. Telomerase positive extract control: add 2 µL of positive control cell extract at a concentration of 500 cells/µL.
   e. Minus telomerase control: add 2 µL of CHAPS Lysis Buffer.
   f. No template control: add 2µL of nuclease free water
   g. PCR amplification control: add 2 µL experimental sample to Control Reaction Mix primed with 0.004 amol TSK Template

PCR Amplification

1. Place the tubes in the thermocycler block.
2. Set-up the real-time experiment to include the following PCR parameters.

30°C	30 min	1 cycle
95°C	2.0 min	1 cycle
**45 cycles**		
94°C	15 sec	
59°C	60 sec	
*45°C	10 sec	

* Temperature and stage where the Real-time fluorescent data should be collected

Note: *These PCR conditions should work on most thermocyclers, but may need to be tested empirically for the specific machine being used. Data collection can also be set for the* 59°C step. *See Sec. IV.* Troubleshooting*.*

Real-time Fluorescence Data

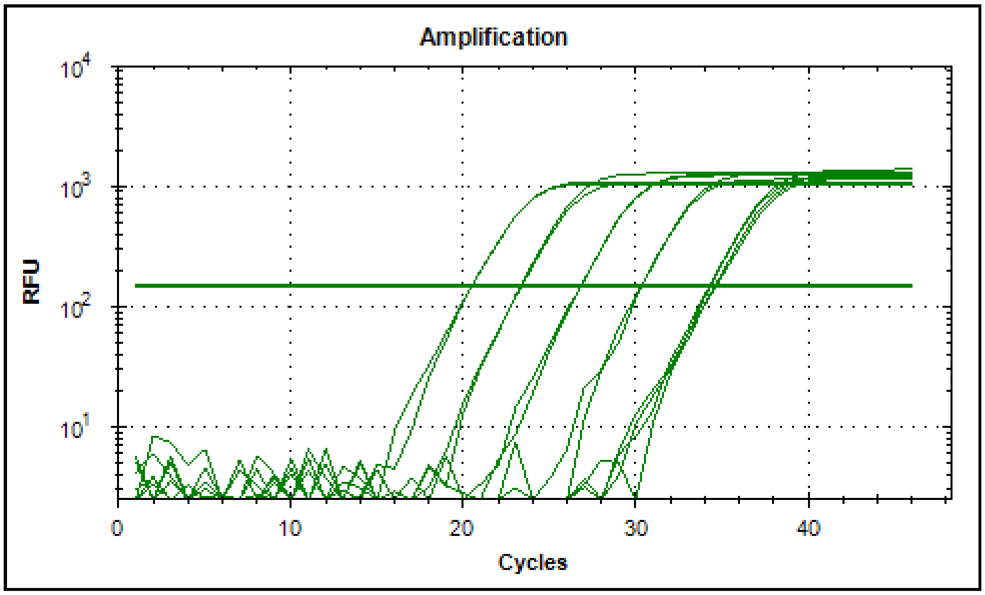

Figure 3: Ten fold serial dilutions of TSR8 Quantitation Control from 40 amoles to 0.04 amoles/well and NTC acquired using an BioRad CFX96™ Real-Time PCR Detection System.

Data Analysis

The following is an example of telomerase activity quantification using fluorometric detection. To obtain valid quantitative results using the TRAPeze® RT Telomerase Detection Kit, the production of a standard curve using the TSR8 template and the inclusion of the assay controls are necessary (see Sec. III. *Protocol, Experimental Design*).

Table 4: Typical Data for the Generation of the TSR8 Standard Curves (Figure 3, 4)

TSR8 Dilutions	Copies TSR8	Log	Experimental Avg. Ct
40 amoles	24,088,000	7.38	20.4
4 amoles	2,408,800	6.38	23.3
0.4 amoles	240,880	5.38	26.8
0.04 amoles	24,080	4.38	30.3

Logarithmic Plot

a. Calculate the $\log_{10}$ of the amoles per well for each reaction (Table 4).

b. Plot each datapoint based on the Ct measurements of the reactions that were performed with the TSR8 dilutions using real time instrument software, a standard graphing program or Microsoft Excel®. Plot the $\log_{10}$ on the X-axis against the average Ct value of the corresponding concentration of TSR8 on the Y-axis. (See Figure 4).

c. Fit datapoints to a linear regression plot using a curve fitting option such as Add Trendline in Microsoft Excel®.

d. The linear equation derived from the data curve fit is used to extrapolate arbitrary telomerase units relative to the TSR8 amplification using the Experimental Sample average Ct values.

e. Telomerase activity from each sample can then be normalized by performing a similar procedure to data derived using 5X TRAPeze® RT Control Reaction Mix and TSK template and relative TSK units derived from a TSK standard curve (Optional). The TSR8 relative unit value can then be divided by the sample specific TSK relative unit value to provide for PCR inhibitor normalization.

Figure 4: Logarithmic Plot of the TSR8 Standard Curve

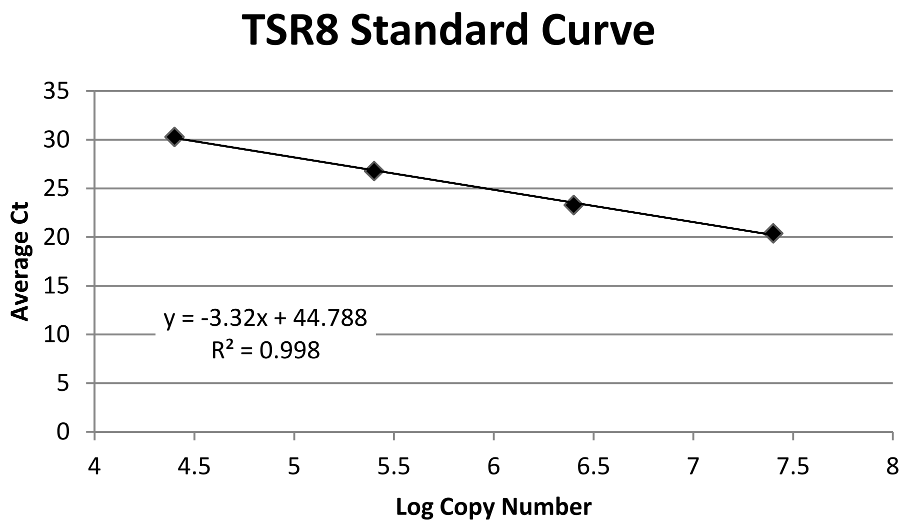

The TRAPeze® RT Kit assay was performed using 10 –fold dilutions of TSR8. Fluorescence was measured using an BioRad CFX96™ Real-Time PCR Detection System.

Sensitivity of the Assay

Sensitivity

Under the conditions described in this manual, telomerase activity in an extract from 50 telomerase-positive control cells can be detected using a real-time fluorescence capable thermocycler. The TSR8 quantitative control can be detected above background at a dilution of 0.04 amols (see Sec. III. *Protocol, TRAPeze® RT Telomerase Detection Kit*).

Note: *The assay sensitivity may vary depending on the instrument utilized. The number of PCR amplification cycles may be increased if necessary. The dilution of TSR8 quantitation control template may be adjusted slightly should experimental samples be detected outside the range of the suggested TSR8 dilution series amplification.*

IV. Troubleshooting

The following are the most commonly encountered problems with the TRAPeze® RT Telomerase detection.

No significant increase in fluorescence signal is observed for the TSR8 quantitation controls, the TSK normalization controls or telomerase-positive samples.

1. Potential problem: PCR amplification is not initiated.

Recommendations:
Check the TRAPeze® RT Kit assay components. Were the 5X TRAPeze® RT Reaction Mix and the Taq polymerase included in the appropriate amounts?
Check the thermocycler for proper temperature and time settings. Is the thermocycler cycling at 94°C / 15 seconds, 59°C / 60 seconds and 45°C / 10 seconds for 45 cycles with data collection selected for FAM in the 45°C stage?
The optimal annealing temperature may need to be tested empirically for each thermocycler.
Check the Taq polymerase to see if it is active. Confirm that a cloned, hot start Taq polymerase was used.

2. Potential Problem: Optically clear tubes were not used for measurements with the fluorescent plate reader.

Recommendations:
1. Perform the TRAPeze® RT Kit assay with optically clear amplification tubes and caps.

Fluorescein signal of the telomerase-positive control cell extract is low.

1. Potential problem: Telomerase activity is not initiated. Possible RNase contamination exists if a positive fluorescence signal is only observed with the TSR8 or TSK controls.

Recommendations:
1. Always use RNase- free tips, tubes and solutions.
2. Use a fresh aliquot of 5X TRAPeze® RT Reaction Mix, taking extra precautions to prevent RNase contamination.
3. Telomerase is heat-sensitive; make sure that the extraction and TRAPeze® RT reaction setup is performed at a temperature below 25°C.
4. Add RNase inhibitor into the CHAPS Lysis Buffer (see Sec. III. *Protocol, Extract Preparation*), taking extra precautions to prevent RNase contamination.
5. Always use a clean labcoat and gloves. Keep the TRAPeze® RT Telomerase Detection Kit Station Setup (Area 1) and telomerase extraction areas clean with bleach and alcohol. (See Sec. V. *Appendix, TRAPeze® RT Telomerase Detection Kit Station Setup (Area 1)*).

All reaction samples and the minus telomerase control (control 2) show a positive fluorescein signal.

1. Potential problems: PCR carry-over contamination.

Recommendations:
1. Use fresh aliquots of every component of the assay (5X TRAPEZE® RT Reaction Mix, Taq polymerase and PCR grade water).
2. Follow the recommendations described in Problem B above and in Sec. V. *Appendix, Laboratory Setup and Precautions*. The PCR tube racks are the most likely source of PCR carry-over contamination. Decontaminate the racks as described in Sec. V. *Appendix, TRAPeze® RT Telomerase Detection Kit Station Setup (Area1)*.

A high fluorescein signal is observed with heat-treated extracts. Extracts are not heat sensitive.

Potential problems: Insufficient heat inactivation of the extracts, primer-dimer PCR artifacts, or contamination of the extract with TRAPeze® RT products.

Recommendations:
1. Check the temperature of the heat block or water bath used for heat inactivation of the extract.
2. Repeat the assay. If the problem persists, it is likely that the extract has a PCR carry-over contamination.
3. Make and test the new extract, taking extra precaution to prevent PCR carryover contamination.
4. Tumor extracts containing a relatively high protein concentration may produce PCR artifacts. The presence of excess proteins in the reaction mixture may result in non-specific PCR amplification products. Dilute the extracts and repeat the analysis.

The data on replicate samples are inconsistent.

Potential problem: Primer-dimer PCR artifacts.

As is anticipated with a PCR-based assay, some unavoidable PCR artifacts are expected even when the optimal assay conditions are employed. Though occurring at a low frequency, these artifacts are most often observed in reactions with minimal telomerase activity. Primer dimers are readily apparent when analyzing the NTC control, and most often are detected at cycles below the limits of sensitivity of the assay.

Recommendations:
1. Recheck the PCR parameters and repeat the assay.
2. Decrease the number of PCR cycles below 45 cycles.
3. Analyze the TRAPeze® RT reaction products by non-denaturing PAGE or high percentage agarose gels (optional). Primer/dimer artifacts are easily distinguishable from genuine telomerase products after samples have undergone electrophoresis through a polyacrylamide gel.
4. Tumor extracts containing a relatively high protein concentration may produce PCR artifacts. The presence of excess proteins in the reaction mixture may result in non-specific PCR amplification products. Dilute the extracts and repeat the analysis.

Minimal TRAPeze® RT Kit assay signals observed for samples where telomerase activity is anticipated.

1. Potential Problem: The cell/tissue extract contains an inhibitor of Taq polymerase.

Recommendations:
1. Dilute the extract 5-, 25- and 125- fold with CHAPS Lysis Buffer, then reanalyze. Sometimes, positive telomerase activity can be detected in the diluted extract that cannot be detected in more concentrated extracts.
2. If higher sensitivity is required with diluted extract refer to Sec. V. *Appendix, Enhancing Detection Sensitivity*.
3. To check for the presence of inhibitor(s), create a "mixed sample" by adding the telomerase-positive cell extract (prepared from cells provided in the kit) to the sample extracts and then perform the TRAPeze® RT Kit assay. If inhibitor(s) of Taq polymerase are present in the sample extracts, the signal observed in the "mixed sample" will be decreased substantially compared to those in the "telomerase-positive cell extract only" sample.

2. Potential Problem: Possible presence of RNase in the sample extracts.

Recommendations:
4. Refer to Problem #2 above.

V. Appendix

Laboratory Setup and Precautions

One of the most important considerations when performing the TRAPeze® RT Kit assay is the environment where the initial reaction mixtures are set up. To prevent false-negative and false-positive results, the ideal environment is free of the contaminants listed below.

1. TSR8 control template
2. Ribonucleases
3. Amplified PCR DNA products

Some sources of TSR8 control template and PCR product contamination are:
1. gel box and buffer
2. contaminated pipettes and tips
3. tube racks
4. notebooks
5. lab coats

6. any other item exposed to amplified PCR products.

Some sources of RNase contamination are:

1. solutions and tubes not treated with an RNase inhibitor
2. any equipment handled without gloves.

The following precautions should be followed in all steps of the assay protocol including the **telomerase extraction** and the **TRAP**eze® **RT Kit assay setup**.

1. **Always use a designated set of pipettes exclusively for pipetting TSR8 control template, and make sure to not use these pipettes in any other part of the assay.** Always use aerosol resistant tips (RNase free). Always wear gloves.
2. Use the H_2O provided in the kit or DEPC treated H_2O for all solutions, aliquot the solutions in small amounts, and use fresh aliquots as "working" solutions which are discarded after use.
3. Keep the assay solutions (5X TRAPeze® RT Reaction Mix, CHAPS Lysis Buffer, dH_2O, Taq polymerase, etc.) separate from other reagents in the lab.
4. Post amplification TRAP procedures should never be carried out near the TRAPeze® RT Kit assay preparative areas.
5. Decontaminate the PCR tube racks with 10% bleach and UV irradiation after each use.
6. The optimal working environment partitions TRAPeze® RT Kit procedures into three areas.

Figure 5: TRAPeze® RT Telomerase Detection Kit Station Setup

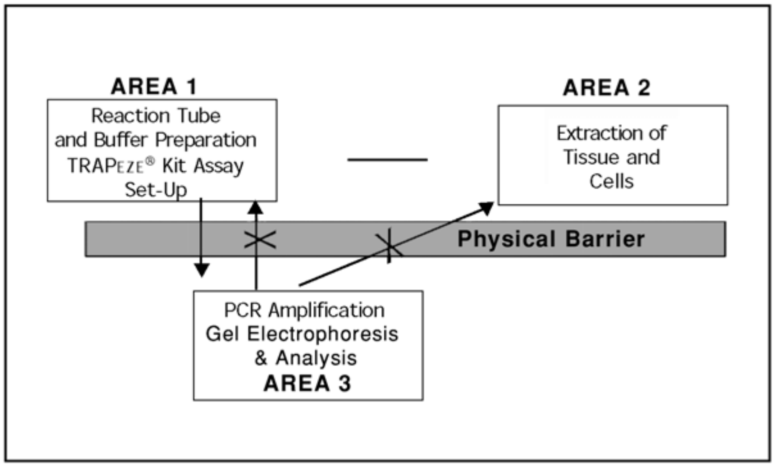

To minimize the potential for carryover contamination, there should be a physical separation of the preparative areas (Areas 1 and 2) from the PCR amplification and detection area (Area 3, see Figure 5). The ideal setup employs separate rooms. If the same room must be used, then a TRAPeze® RT Telomerase Detection Kit assay station setup should be adopted with a clear division between the preparation area and the PCR amplification/ detection area. Another option is to separate the tasks between personnel: one individual carries out the preparation of the extract and TRAPeze® RT Kit assay set up, and another performs the analysis of the amplified products. It is mandatory that no amplified products or equipment exposed to the amplified products (Area 3) enter the preparative areas (Area 1 and 2). Optimally, Areas 1 and 2 should be in separate rooms or spaces. However, this is not as critical as separating Areas 1 and 2 from Area 3. Usually, preparation of tissues and cell extracts are performed in a laminar flow hood with appropriate sterile protocols, so the division between Area 1 and Area 2 (tissue culture hood) is convenient.

TRAPeze® RT Telomerase Detection Kit Station Setup (Area 1)

Laboratory personnel can easily be contaminated with PCR products when carrying out routine manipulations such as opening tubes, pipetting PCR products, or discarding gel buffer. DNA may remain on the person for many days. To avoid this source of contamination, a positive air displacement hood that blows in filtered air over the workspace toward the investigator works well. Separate solutions, pipettes, tubes, and tips should always be used and kept inside the hood. The work space should be wiped with 10% bleach prior to set up of the reaction, and the hood should be routinely UV irradiated (shortwave) when not in use.

Once every 1-3 weeks, the barrels of pipettes should be soaked in 10% bleach, even if aerosol resistant tips are used. The investigator should always wear gloves and use clean lab coats or disposable sleeves which should be changed every week.

Excitation and Emission Filters

For the direct detection of fluorescein and sulforhodamine, it is essential to utilize the appropriate excitation and emission filters with the fluorescence plate reader. It is recommended that >20 nm should separate the upper limit (maximum λ) of the excitation filter bandwidth and the lower limit (minimum λ) of the emission filter bandwidth (λ= wavelength). This will ensure that none of excitation light will contribute to the emission signal (all crosstalk will be eliminated).

Table 5: Middle λ of Filter Bandwidth

	Excitation	Emission
Fluorescein	485 nm	535 nm

V. References

References Cited in the Manual

1. Blackburn, E.H. (1984) The molecular structure of centromeres and telomeres. *Annu. Rev. Biochem.* **53:**163-94.
2. Blackburn, E.H. (1991) Structure and function of telomeres. *Nature* **350:**569-573.
3. Zakitan, V.A. (1989) Structure and function of telomeres. *Ann. Rev. Genet.* **23:**579.
4. Harley, C.B., A.B. Futcher and C.W. Greider. (1990) Telomeres shorten during aging of human fibroblasts. *Nature* **345:**458-460.
5. Hastie, N. D., M. Dempster, M.G. Dunlop, A.M. Thompson, D.K. Green and R.C. Allshire. (1990) Telomere reduction in human colorectal carcinoma and with aging. *Nature* **346:**866-868.
6. Lindsey, J., N.I. McGill, L.A. Lindsey, D.K. Green and H.J Cooke. (1991) In vivo loss of telomeric repeats with age in humans. *Mutat. Res.* **256:**45-48.
7. Counter, C. M. et al. (1992) Telomerase shortening associated with chromosome instability is arrested in immortal cells which express telomerase activity. *EMBO J.* **11:**1921.
8. Watson, J. D. (1972) Origin of concatemeric T7 DNA. *Nature New Biol.* **239:**197-201.
9. Olovnikov, A. M. (1973) A theory of marginotomy. The incomplete copying of template margin in enzymic synthesis of polynucleotides and biological significance of the phenomenon. *J. Theor. Biol.* **41:**181-190.
10. Greider, C.W. and E.H. Blackburn. (1989) A telomeric sequence in the RNA of Tetrahymena telomerase required for telomere repeats synthesis. *Nature* **337:**331-337.
11. Morin, G.B. (1989) The human telomere terminal transferase enzyme is a ribonucleoprotein that synthesizes TTAGGG repeats. *Cell* **59:**521-529.
12. Feng, J. et al. (1997) The RNA component of human telomerase. *Science* **269:**1236-1241
13. Nakamura, T. M., G.B. Morin, K.B. Chapman, S.L. Weinrich, W.H. Andrews, J. Lingner, C.B. Harley and T.R. Cech. (1997) Telomerase catalytic subunit homologs from fission yeast and humans. *Science* **277:**955-959.
14. Meyerson, M., C.M. Counter, E.N. Eaton, L.W. Ellisen, P. Steiner, S.D. Caddle, L. Ziaugra, R.L. Beijersbergen, M.J. Davidoff, Q. Liu, S. Bacchetti, D.A. Haber and R.A. Weinberg. (1997) hEST2, the putative telomerase catalytic subunit gene, is up-regulated in tumor cells and during immortalization. *Cell* **90:**785-795.
15. Kim, N.W., M.A. Piatyszek, K.R. Prowse, C.B. Harley, M.D. West, P.L. Ho, G.M. Coviello, W.E. Wright, S.L. Weinrich and J.W. Shay. (1994) Specific association of human telomerase activity with immortal cells and cancer. *Science* **266:**2011-2015.
16. Shay, J.W. and W.E. Wright. (1996) Telomerase activity in human cancer. *Current Opinion in Oncology* **8:**66-71.
17. Counter, C. M., F.M. Botelho, P. Wang, C.B. Harley and S. Bacchetti. (1994) Stabilization of short telomeres and telomerase activity accompany immortalization of Epstein-Barr virus-transformed human B lymphocytes. *J. Virol.* **68:**3410-3414.
18. Harley, C.B. (1991) Telomere loss: mitotic clock or genetic time bomb? *Mutat. Res.* **256:**271-282.
19. Greider, C.W. (1990) Telomeres, telomerase and senescence. *BioEssays* **12:**363-369.

20. Harley, C. B., N.W. Kim, K.R. Prouse, S.L. Weinrich, K.S. Hirsch, M.D. West, S. Bachetti, H.W. Hirte, C.M. Counter, C.W. Greider, et al. (1994) Telomerase, cell immortality and cancer. *Cold Spring Harbor Symp. On Quant. Biol.* **59:**307-15.
21. Bodnar, A.G., M. Ouellette, M. Frolkis, S.E. Holt, C-P. Chiu, G.B. Morin, C.B. Harley, J.W. Shay, S. Lichsteiner and W.E. Wright. (1998) Extension of life-span by introduction of telomerase into normal human cells. *Science* **279:**349-352.
22. Piatyszek, M. A. et al. (1995) Detection of telomerase activity in human cells and tumors by telomeric repeat amplification protocol (TRAP). *Methods in Cell Science* **17:**1.
23. Sommerfeld, H.J., A.K. Meeker, M.A. Piatyszek, G.S. Bora, J.W. Shay and D.S. Coffey. (1996) Telomerase activity: a prevalent marker of malignant human prostate tissue. *Cancer Res.* **56:**218-222.
24. Broccoli, D., J.W. Young, and T. de Lange. (1995) Telomerase activity in normal and malignant hematopoietic cells. *Proc. Natl. Acad. Sci. USA* **92:**9082-9086.
25. Counter, C.M., H.W. Hirte, S. Bachetti and C.B. Harley. (1994) Telomerase activity in human ovarian carcinoma. *Proc. Natl. Acad. Sci. USA* **91:**2900-2904.
26. Hiyama, E., K. Hiyama, T. Yokoyama, Y. Matsuura, M.A. Piatyszek and J.W. Shay. (1995) Correlating telomerase activity levels with human neuroblastoma outcomes. *Nature Medicine* **1:**249-255.
27. Hiyama, K., E. Hiyama, S. Ishioka, M. Yamakido, K. Inai, A.F. Gazdar, M.A. Piatyszek, and J.W. Shay. (1995) Telomerase activity in small-cell and non-small-cell lung cancers. *J. Natl. Cancer Inst.* **87:**895-902.
28. Mehle, C., M.A. Piatyszek, B. Ljungberg, J.W. Shay and G. Roos. (1996) Telomerase activity in human renal cell carcinoma. *Oncogene* **13:**161-166.
29. Li, Z.H., R. Salovaara, L.A. Aaltonen and D. Shibata. (1996) Telomerase activity is commonly detected in hereditary nonpolyposis colorectal cancers. *Am. J. Pathol.* **148:**1075-1079.
30. Shay, J.W. and S. Bacchetti. (1997) A survey of telomerase activity in human cancer. *Eur. J. Cancer* **33:**787-791.
31. Nelson, N.J. (1996) Researchers debate clinical role of telomerase. *J. Natl. Cancer Inst.* **88:**1021-1023.
32. Morin, G.B. (1991) Recognition of chromosome truncation site associated with a-thalassaemia by human telomerase. *Nature* **353:**454-456.
33. Nazarenko, I.A., Bhatnagar, S. and R.J. Hohman. (1997) A closed tube format for amplification and detection of DNA based on energy transfer. *Nucleic Acids Research* **25 (12):**2516-2521
34. Uehara, H., Nardone, G., Nazarenko, I.A and R.J. Hohman. (1999) Detection of telomerase activity utilizing energy transfer primers: comparison with gel- and ELISA-based detection. *Biotechniques* **26 (3):**552-558.

Disclaimers

SYBR® is a registered trademark of Molecular Probes.

Microsoft Excel® is a registered trademark of Microsoft Corporation.

Titanium™ is a trademark of Becton Dickinson, Inc.

ABI Prism®7700/7900 is a registered trademark of Applied Biosystems.

iCycler® and MJ Opticon™ are trademarks of Bio-Rad Laboratories.

Stratagene MX® 4000 is a registered trademark of Stratagene®.

This product is covered under U.S. Patent # 5,866,336 and is sold for research use only.

Licensed from Geron Corporation under U.S. Patent #5,629,154 and other patents pending. No license to use the TRAP assay for clinical diagnostic purposes or to discover drugs that modulate telomerase activity is granted by implication or estoppel by the purchase of this product.

The polymerase chain reaction ("PCR") is covered by one or more of the following U.S. Patents: nos. 4,683,202; 4,899,818 issued to Cetus Corporation and owned and licensed by Hoffmann-LaRoche Molecular Systems, Inc. Purchase of a CHEMICON or Serologicals' PCR-related product does not convey a license to use the PCR process covered by these patents. Purchasers of these products must obtain a license to use the PCR process before performing PCR.

All trademarks, unless otherwise noted, are the property of Serologicals Royalty Company or Chemicon International, Inc.

Warranty

EMD Millipore Corporation ("EMD Millipore") warrants its products will meet their applicable published specifications when used in accordance with their applicable instructions for a period of one year from shipment of the products. **EMD MILLIPORE MAKES NO OTHER WARRANTY, EXPRESSED OR IMPLIED. THERE IS NO WARRANTY OF MERCHANTABILITY OR FITNESS FOR A PARTICULAR PURPOSE.** The warranty provided herein and the data, specifications and descriptions of EMD Millipore products appearing in EMD Millipore's published catalogues and product literature may not be altered except by express written agreement signed by an officer of EMD Millipore. Representations, oral or written, which are inconsistent with this warranty or such publications are not authorized and if given, should not be relied upon.

In the event of a breach of the foregoing warranty, EMD Millipore Corporation's sole obligation shall be to repair or replace, at its option, the applicable product or part thereof, provided the customer notifies EMD Millipore Corporation promptly of any such breach. If after exercising reasonable efforts, EMD Millipore Corporation is unable to repair or replace the product or part, then EDM Millipore shall refund to the Company all monies paid for such applicable Product. **EMD MILLIPORE CORPORATION SHALL NOT BE LIABLE FOR CONSEQUENTIAL, INCIDENTAL, SPECIAL OR ANY OTHER DAMAGES RESULTING FROM ECONOMIC LOSS OR PROPERTY DAMAGE SUSTAINED BY ANY COMPANY CUSTOMER FROM THE USE OF ITS PRODUCTS.**

Unless otherwise stated in our catalog or other company documentation accompanying the product(s), our products are intended for research use only and are not to be used for any other purpose, which includes but is not limited to, unauthorized commercial uses, in vitro diagnostic uses, ex vivo or in vivo therapeutic uses or any type of consumption or application to humans or animals.

(c) 2009 - 2014: Merck KGaA, Darmstadt. All rights reserved. No part of these works may be reproduced in any form without permission in writing.

Cat No. S7710
