# Supplementary material for: The consequences of a high-calorie diet background before calorie restriction on skeletal muscles in a mouse model
Source: Aging (Albany NY). 2021 Jun 24;13(12):16834–58. doi: 10.18632/aging.203237 (PMC8266348; doi:10.18632/aging.203237)
Supplement: Supplementary Material 3 [file aging-13-203237-s007.pdf]

Mouse NAD-Dependent Deacetylase Sirtuin-1 (Sirt1) ELISA Kit

Catalog Number. CSB-E16187m

For the quantitative determination of mouse NAD-dependent deacetylase sirtuin-1 (Sirt1) concentrations in serum, plasma, tissue homogenates.

This package insert must be read in its entirety before using this product.

If You Have Problems

Technical Service Contact information

Phone: 86-27-87582341
Fax: 86-27-87196150
Email: tech@cusabio.com
Web: www.cusabio.com

In order to obtain higher efficiency service, please ready to supply the lot number of the kit to us (found on the outside of the box).

PRINCIPLE OF THE ASSAY

This assay employs the quantitative sandwich enzyme immunoassay technique. Antibody specific for Sirt1 has been pre-coated onto a microplate. Standards and samples are pipetted into the wells and any Sirt1 present is bound by the immobilized antibody. After removing any unbound substances, a biotin-conjugated antibody specific for Sirt1 is added to the wells. After washing, avidin conjugated Horseradish Peroxidase (HRP) is added to the wells. Following a wash to remove any unbound avidin-enzyme reagent, a substrate solution is added to the wells and color develops in proportion to the amount of Sirt1 bound in the initial step. The color development is stopped and the intensity of the color is measured.

DETECTION RANGE

0.156 ng/ml-10 ng/ml.

SENSITIVITY

The minimum detectable dose of mouse Sirt1 is typically less than 0.039 ng/ml.

The sensitivity of this assay, or Lower Limit of Detection (LLD) was defined as the lowest protein concentration that could be differentiated from zero. It was determined the mean O.D value of 20 replicates of the zero standard added by their three standard deviations.

SPECIFICITY

This assay has high sensitivity and excellent specificity for detection of mouse Sirt1. No significant cross-reactivity or interference between mouse Sirt1 and analogues was observed.

Note: Limited by current skills and knowledge, it is impossible for us to complete the cross-reactivity detection between mouse Sirt1 and all the analogues, therefore, cross reaction may still exist.

PRECISION

Intra-assay Precision (Precision within an assay): CV%<8%

Three samples of known concentration were tested twenty times on one plate to assess.

Inter-assay Precision (Precision between assays): CV%<10%

Three samples of known concentration were tested in twenty assays to assess.

LIMITATIONS OF THE PROCEDURE

- **FOR RESEARCH USE ONLY. NOT FOR USE IN DIAGNOSTIC PROCEDURES.**
- The kit should not be used beyond the expiration date on the kit label.
- Do not mix or substitute reagents with those from other lots or sources.
- If samples generate values higher than the highest standard, dilute the samples with Sample Diluent and repeat the assay.
- Any variation in Sample Diluent, operator, pipetting technique, washing technique, incubation time or temperature, and kit age can cause variation in binding.
- This assay is designed to eliminate interference by soluble receptors, binding proteins, and other factors present in biological samples. Until all factors have been tested in the Immunoassay, the possibility of interference cannot be excluded.

MATERIALS PROVIDED

Reagents	Quantity
Assay plate (12 x 8 coated Microwells)	1(96 wells)
Standard (Freeze dried)	2
Biotin-antibody (100 x concentrate)	1 x 120 μl
HRP-avidin (100 x concentrate)	1 x 120 μl
Biotin-antibody Diluent	1 x 15 ml
HRP-avidin Diluent	1 x 15 ml
Sample Diluent	1 x 50 ml
Wash Buffer (25 x concentrate)	1 x 20 ml
TMB Substrate	1 x 10 ml
Stop Solution	1 x 10 ml
Adhesive Strip (For 96 wells)	4
Instruction manual	1

STORAGE

<table>
<tr><td>Unopened kit</td><td colspan="2">Store at 2 - 8°C. Do not use the kit beyond the expiration date.</td></tr>
<tr><td rowspan="11">Opened kit</td><td>Coated assay plate</td><td>May be stored for up to 1 month at 2 - 8°C. Try to keep it in a sealed aluminum foil bag, and avoid the damp.</td></tr>
<tr><td>Standard</td><td rowspan="3">May be stored for up to 1 month at 2 - 8° C. If don’t make recent use, better keep it store at -20°C.</td></tr>
<tr><td>Biotin-antibody</td></tr>
<tr><td>HRP-avidin</td></tr>
<tr><td>Biotin-antibody Diluent</td><td rowspan="7">May be stored for up to 1 month at 2 - 8°C.</td></tr>
<tr><td>HRP-avidin Diluent</td></tr>
<tr><td>Sample Diluent</td></tr>
<tr><td>Wash Buffer</td></tr>
<tr><td>TMB Substrate</td></tr>
<tr><td>Stop Solution</td></tr>
</table>

***Provided this is within the expiration date of the kit.**

OTHER SUPPLIES REQUIRED

- Microplate reader capable of measuring absorbance at 450 nm, with the correction wavelength set at 540 nm or 570 nm.
- An incubator which can provide stable incubation conditions up to 37°C±0.5°C.
- Squirt bottle, manifold dispenser, or automated microplate washer.
- Absorbent paper for blotting the microtiter plate.
- 100ml and 500ml graduated cylinders.
- Deionized or distilled water.
- Pipettes and pipette tips.
- Test tubes for dilution.

PRECAUTIONS

The Stop Solution provided with this kit is an acid solution. Wear eye, hand, face, and clothing protection when using this material.

SAMPLE COLLECTION AND STORAGE

- **Serum** Use a serum separator tube (SST) and allow samples to clot for two hours at room temperature or overnight at 4°C before centrifugation for 15 minutes at 1000 × g. Remove serum and assay immediately or aliquot and store samples at -20°C or -80°C. Avoid repeated freeze-thaw cycles.
- **Plasma** Collect plasma using EDTA, or heparin as an anticoagulant. Centrifuge for 15 minutes at 1000 × g at 2 - 8°C within 30 minutes of collection. Assay immediately or aliquot and store samples at -20°C or -80°C. Avoid repeated freeze-thaw cycles. Centrifuge the sample again after thawing before the assay.
- **Tissue Homogenates** 100mg tissue was rinsed with 1X PBS, homogenized in 1 ml of 1X PBS and stored overnight at -20°C. After two freeze-thaw cycles were performed to break the cell membranes, the homogenates were centrifuged for 5 minutes at 5000 x g, 2 - 8°C. The supernate was removed and assayed immediately. Alternatively, aliquot and store samples at -20°C or -80°C. Centrifuge the sample again after thawing before the assay. Avoid repeated freeze-thaw cycles.

Note:

1. CUSABIO is only responsible for the kit itself, but not for the samples consumed during the assay. The user should calculate the possible amount of the samples used in the whole test. Please reserve sufficient samples in advance.
2. Samples to be used within 5 days may be stored at 2-8°C, otherwise samples must be stored at -20°C (≤1month) or -80°C (≤2month) to avoid loss of bioactivity and contamination.
3. Grossly hemolyzed samples are not suitable for use in this assay.
4. If the samples are not indicated in the manual, a preliminary experiment to determine the validity of the kit is necessary.
5. Please predict the concentration before assaying. If values for these are not within the range of the standard curve, users must determine the optimal sample dilutions for their particular experiments.
6. Tissue or cell extraction samples prepared by chemical lysis buffer may cause unexpected ELISA results due to the impacts of certain chemicals.
7. Owing to the possibility of mismatching between antigen from other resource and antibody used in our kits (e.g., antibody targets conformational epitope rather than linear epitope), some native or recombinant proteins from other manufacturers may not be recognized by our products.
8. Influenced by the factors including cell viability, cell number and also sampling time, samples from cell culture supernatant may not be detected by the kit.
9. Fresh samples without long time storage are recommended for the test. Otherwise, protein degradation and denaturalization may occur in those samples and finally lead to wrong results.

REAGENT PREPARATION

Note:

- **Kindly use graduated containers to prepare the reagent. Please don't prepare the reagent directly in the Diluent vials provided in the kit.**
- Bring all reagents to room temperature (18-25°C) before use for 30min.
- Prepare fresh standard for each assay. Use within 4 hours and discard after use.
- Making serial dilution in the wells directly is not permitted.
- Please carefully reconstitute Standards according to the instruction, and avoid foaming and mix gently until the crystals have completely dissolved. To minimize imprecision caused by pipetting, use small volumes and ensure that pipettors are calibrated. It is recommended to suck more than 10μl for once pipetting.
- Distilled water is recommended to be used to make the preparation for reagents. Contaminated water or container for reagent preparation will influence the detection result.

1. **Biotin-antibody (1x) -** Centrifuge the vial before opening.
   Biotin-antibody requires a 100-fold dilution. A suggested 100-fold dilution is 10 μl of **Biotin-antibody** + 990 μl of **Biotin-antibody Diluent**.

2. **HRP-avidin (1x) -** Centrifuge the vial before opening.
   HRP-avidin requires a 100-fold dilution. A suggested 100-fold dilution is 10 μl of **HRP-avidin** + 990 μl of **HRP-avidin Diluent**.

3. **Wash Buffer(1x)-** If crystals have formed in the concentrate, warm up to room temperature and mix gently until the crystals have completely dissolved. Dilute 20 ml of Wash Buffer Concentrate (25 x) into deionized or distilled water to prepare 500 ml of Wash Buffer (1 x).

4. **Standard**

Centrifuge the standard vial at 6000-10000rpm for 30s.

Reconstitute the **Standard** with 1.0 ml of **Sample Diluent**. Do not substitute other diluents. This reconstitution produces a stock solution of 10 ng/ml. Mix the standard to ensure complete reconstitution and allow the standard to sit for a minimum of 15 minutes with gentle agitation prior to making dilutions.

Pipette 250 μl of **Sample Diluent** into each tube (S0-S6). Use the stock solution to produce a 2-fold dilution series (below). Mix each tube thoroughly before the next transfer. The undiluted Standard serves as the high standard (10 ng/ml). **Sample Diluent** serves as the zero standard (0 ng/ml).

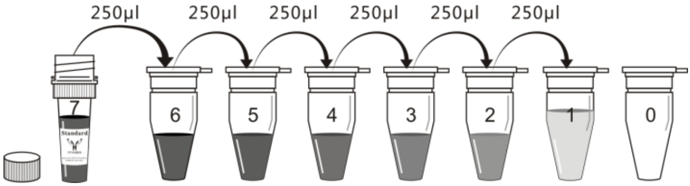

Tube	S7	S6	S5	S4	S3	S2	S1	S0
ng/ml	10	5	2.5	1.25	0.625	0.312	0.156	0

ASSAY PROCEDURE

Bring all reagents and samples to room temperature before use. Centrifuge the sample again after thawing before the assay. It is recommended that all samples and standards be assayed in duplicate.

1. Prepare all reagents, working standards, and samples as directed in the previous sections.
2. Refer to the Assay Layout Sheet to determine the number of wells to be used and put any remaining wells and the desiccant back into the pouch and seal the ziploc, store unused wells at 4°C.
3. Add 100µl of standard and sample per well. Cover with the adhesive strip provided. Incubate for 2 hours at 37°C. A plate layout is provided to record standards and samples assayed.
4. Remove the liquid of each well, **don’t wash.**
5. Add 100µl of **Biotin-antibody (1x)** to each well. Cover with a new adhesive strip. Incubate for 1 hour at 37°C. (**Biotin-antibody (1x)** may appear cloudy. Warm up to room temperature and mix gently until solution appears uniform.)
6. Aspirate each well and wash, repeating the process two times for a total of three washes. Wash by filling each well with Wash Buffer (200µl) using a squirt bottle, multi-channel pipette, manifold dispenser, or autowasher, and let it stand for 2 minutes, complete removal of liquid at each step is essential to good performance. After the last wash, remove any remaining wash Buffer by aspirating or decanting. Invert the plate and blot it against clean paper towels.
7. Add 100µl of **HRP-avidin (1x)** to each well. Cover the microtiter plate with a new adhesive strip. Incubate for 1 hour at 37°C.
8. Repeat the aspiration/wash process for five times as in step 6.
9. Add 90µl of **TMB Substrate** to each well. Incubate for 15-30 minutes at 37°C. **Protect from light.**
10. Add 50µl of **Stop Solution** to each well, gently tap the plate to ensure thorough mixing.

11. Determine the optical density of each well within 5 minutes, using a microplate reader set to 450 nm. If wavelength correction is available, set to 540 nm or 570 nm. Subtract readings at 540 nm or 570 nm from the readings at 450 nm. This subtraction will correct for optical imperfections in the plate. Readings made directly at 450 nm without correction may be higher and less accurate.

Note:

1. The final experimental results will be closely related to validity of the products, operation skills of the end users and the experimental environments.
2. Samples or reagents addition: Please use the freshly prepared Standard. Please carefully add samples to wells and mix gently to avoid foaming. Do not touch the well wall as possible. For each step in the procedure, total dispensing time for addition of reagents or samples to the assay plate should not exceed 10 minutes. This will ensure equal elapsed time for each pipetting step, without interruption. Duplication of all standards and specimens, although not required, is recommended. To avoid cross-contamination, change pipette tips between additions of each standard level, between sample additions, and between reagent additions. Also, use separate reservoirs for each reagent.
3. Incubation: To ensure accurate results, proper adhesion of plate sealers during incubation steps is necessary. Do not allow wells to sit uncovered for extended periods between incubation steps. Once reagents have been added to the well strips, DO NOT let the strips DRY at any time during the assay. Incubation time and temperature must be observed.
4. Washing: The wash procedure is critical. Complete removal of liquid at each step is essential to good performance. After the last wash, remove any remaining Wash Solution by aspirating or decanting and remove any drop of water and fingerprint on the bottom of the plate. Insufficient washing will result in poor precision and falsely elevated absorbance reading. When using an automated plate washer, adding a 30 second soak period following the addition of wash buffer, and/or rotating the plate 180 degrees between wash steps may improve assay precision.

5. Controlling of reaction time: Observe the change of color after adding TMB Substrate (e.g. observation once every 10 minutes), TMB Substrate should change from colorless or light blue to gradations of blue. If the color is too deep, add Stop Solution in advance to avoid excessively strong reaction which will result in inaccurate absorbance reading.
6. TMB Substrate is easily contaminated. TMB Substrate should remain colorless or light blue until added to the plate. Please protect it from light.
7. Stop Solution should be added to the plate in the same order as the TMB Substrate. The color developed in the wells will turn from blue to yellow upon addition of the Stop Solution. Wells that are green in color indicate that the Stop Solution has not mixed thoroughly with the TMB Substrate.

ASSAY PROCEDURE SUMMARY

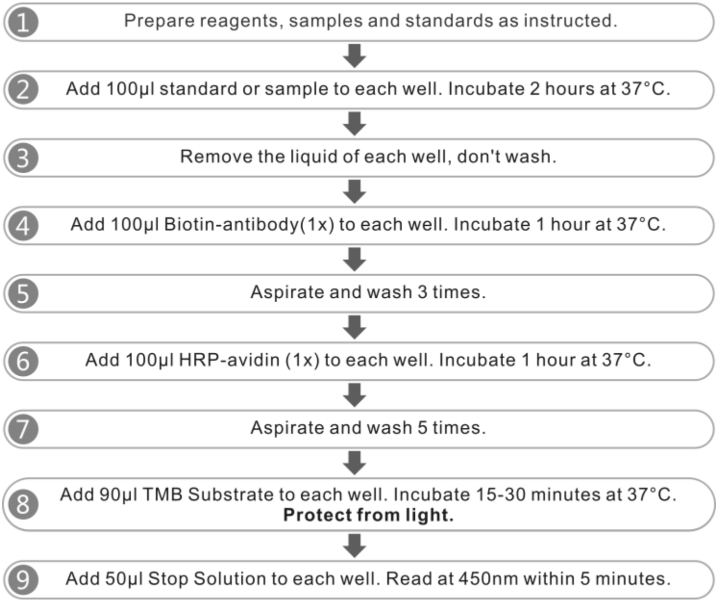

***Please determine whether the sample needs to be diluted or the optimal dilution factor based on preliminary experiment result.**

CALCULATION OF RESULTS

Using the professional soft "Curve Expert" to make a standard curve is recommended, which can be downloaded from our web.

Average the duplicate readings for each standard and sample and subtract the average zero standard optical density.

Create a standard curve by reducing the data using computer software capable of generating a four parameter logistic (4-PL) curve-fit. As an alternative, construct a standard curve by plotting the mean absorbance for each standard on the x-axis against the concentration on the y-axis and draw a best fit curve through the points on the graph. The data may be linearized by plotting the log of the Sirt1 concentrations versus the log of the O.D. and the best fit line can be determined by regression analysis. This procedure will produce an adequate but less precise fit of the data.

If samples have been diluted, the concentration read from the standard curve must be multiplied by the dilution factor.
